# Feeding High-Fat Diet Accelerates Development of Peripheral and Central Insulin Resistance and Inflammation and Worsens AD-like Pathology in APP/PS1 Mice

**DOI:** 10.3390/nu15173690

**Published:** 2023-08-23

**Authors:** Anna Mengr, Veronika Strnadová, Štěpán Strnad, Vladimír Vrkoslav, Helena Pelantová, Marek Kuzma, Thomas Comptdaer, Blanka Železná, Jaroslav Kuneš, Marie-Christine Galas, Andrea Pačesová, Lenka Maletínská

**Affiliations:** 1 Institute of Organic Chemistry and Biochemistry of the Czech Academy of Sciences, Flemingovo nám. 2, Prague 6, 166 10 Prague, Czech Republic; anna.mengr@uochb.cas.cz (A.M.); veronika.strnadova@uochb.cas.cz (V.S.); stepan.strnad@uochb.cas.cz (Š.S.); vladimir.vrkoslav@uochb.cas.cz (V.V.); blanka.zelezna@uochb.cas.cz (B.Ž.); Jaroslav.Kunes@fgu.cas.cz (J.K.); 2 Institute of Microbiology of the Czech Academy of Sciences, Vídeňská 1083, Prague 4, 142 20 Prague, Czech Republic; pelantova@biomed.cas.cz (H.P.); kuzma@biomed.cas.cz (M.K.); 3 University of Lille, Inserm, CHU Lille, CNRS, LilNCog-Lille Neuroscience & Cognition, F-59000 Lille, France; thomas.comptdaer@inserm.fr (T.C.); marie-christine.galas@inserm.fr (M.-C.G.); 4 Institute of Physiology of the Czech Academy of Sciences, Vídeňská 1083, Prague 4, 142 20 Prague, Czech Republic

**Keywords:** insulin resistance, glucose intolerance, inflammation, neuroinflammation, obesity, Alzheimer’s disease, amyloid-β, tau protein, APP/PS1

## Abstract

Alzheimer’s disease (AD) is a progressive brain disorder characterized by extracellular amyloid-β (Aβ) plaques, intracellular neurofibrillary tangles formed by hyperphosphorylated Tau protein and neuroinflammation. Previous research has shown that obesity and type 2 diabetes mellitus, underlined by insulin resistance (IR), are risk factors for neurodegenerative disorders. In this study, obesity-induced peripheral and central IR and inflammation were studied in relation to AD-like pathology in the brains and periphery of APP/PS1 mice, a model of Aβ pathology, fed a high-fat diet (HFD). APP/PS1 mice and their wild-type controls fed either a standard diet or HFD were characterized at the ages of 3, 6 and 10 months by metabolic parameters related to obesity via mass spectroscopy, nuclear magnetic resonance, immunoblotting and immunohistochemistry to quantify how obesity affected AD pathology. The HFD induced substantial peripheral IR leading to central IR. APP/PS1-fed HFD mice had more pronounced IR, glucose intolerance and liver steatosis than their WT controls. The HFD worsened Aβ pathology in the hippocampi of APP/PS1 mice and significantly supported both peripheral and central inflammation. This study reveals a deleterious effect of obesity-related mild peripheral inflammation and prediabetes on the development of Aβ and Tau pathology and neuroinflammation in APP/PS1 mice.

## 1. Introduction

Alzheimer’s disease (AD) is an age-associated neurological disorder, accounting for 60–80% of all dementia cases [1], characterized by the presence of extracellular senile plaques formed by amyloid-β (Aβ) peptide and intracellular neurofibrillary tangles formed by hyperphosphorylated Tau protein as its two main hallmarks [2], as well as reduced glucose metabolism in the brain, neuroinflammation, extensive synaptic loss and neuronal loss in vulnerable brain areas [3,4,5].

Obesity and type 2 diabetes mellitus (T2DM) have been established as risk factors for developing AD; an useful model of human obesity is rodents with diet-induced obesity (DIO) [6]. Only two weeks of high-fat diet (HFD) feeding induced an increase in adipose tissue (AT) and consequently led to hyperleptinemia and leptin resistance, proceeding to hyperglycemia and insulin resistance (IR) [7,8] with impaired insulin signaling and glucose uptake mainly in the muscles and AT [9,10]. Likewise, a decreased capability for glucose uptake occurs in AD brains, thus resembling the conditions of brain IR [9]. Moreover, HFD-induced IR further results in a loss of cognition due to impaired insulin regulation, increased inflammation, mitochondrial dysfunction, increased oxidative stress and apoptosis in the brain [9,11,12]. Interestingly, APP/PS1 mice, a mouse model of amyloidosis that expresses mutated amyloid-β protein precursor (APP) (Swedish mutation, K595N/M596L) and mutated presenilin (PS1) (deltaE9 PS1 exon deletion) [13], were more susceptible to HFD-induced weight gain [14,15,16] accompanied by increased AT mass and leptin levels [16]. Long-term HFD consumption in APP/PS1 males also induced glucose intolerance [17], increased levels of plasma insulin and decreased levels of glucose transporters (GLUT) 2 in the liver and GLUT 4 in skeletal muscle [14,15]. HFD also led to lipid accumulation in the liver and increased serum lipids and cytokines [14,15]. APP/PS1 mice on HFD showed increased levels of Aβ1-40 and Aβ1-42 [14] and subsequently increased levels of Aβ plaques in the brain [18,19]. Further, increased glial fibrillary acidic protein (GFAP) and Ionized calcium-binding adapter molecule 1 (Iba1), both markers of neuroinflammation, were observed.

HFD feeding exacerbated AD parameters and memory impairment in other mouse models of AD, such as in Thy-Tau22 mice, a transgenic mouse model of tauopathy [20,21] or in 3xTg-AD mice, a model combining tauopathy and amyloidosis [22].

To elucidate the mechanisms linking obesity to the development of neurodegeneration, we fed APP/PS1 mice a HFD and observed how increased adiposity, IR, inflammation in the periphery and the brain cause AD-like pathology using different approaches, such as metabolomics, lipidomics, immunoblotting and immunohistochemistry.

## 2. Materials and Methods

### 2.1. Animals

All animal experiments were performed following the ethical guidelines for animal experiments of the Czech Republic Act Nr. 246/1992 and were approved by the Committee for Experiments with Laboratory Animals of Czech Academy of Sciences (CAS).

### 2.2. Study Design and Treatment

**Figure 1:**
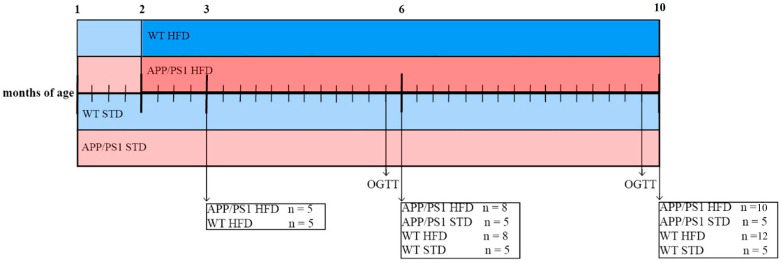
Experimental design of the study. At the age of 2 months, part of the wild-type (WT, *n* = 5–12 mice per group) and APP/PS1 mice (*n* = 5–10 mice per group) were switched to a high-fat diet (HFD); the rest stayed at a standard diet (STD, *n* = 5 mice per group). For characterization, mice were sacrificed at the ages of 3, 6 and 10 months. A week before dissections of 6- and 10-month old mice, oral glucose tolerance tests (OGTTs) were performed.

At the age of 7 weeks, APP/PS1 and their wild-type (WT) controls arrived from the Biotechnology and Biomedicine Centre of the Academy of Sciences and Charles University (BIOCEV) (Vestec, Czech Republic) to the animal facility of the IOCB CAS (with a 12 h light/dark cycle (lights on at 5 a.m.) and temperature of 23 ± 2 °C). The mice were housed 4–5 per cage until the end of the experiment and were given free access to water and food. At the age of 8 weeks, mice were randomly divided into groups of 5–12 animals, and according to Figure 1, they were fed either a standard diet (STD) or a HFD with caloric content percentage values of 13% protein, 60% fat and 27% carbohydrate prepared according to Matyšková et al. [23]. The APP/PS1 and WT mice were characterized at the ages of 3, 6 and 10 months. The body weight (BW) of the mice was monitored once per week. For the following analyses, mice were selected according to their similar BW within the set of animals; the exact number of animals is shown in figure or table legends.

### 2.3. Oral Glucose Tolerance Test (OGTT)

OGTTs were performed on 6 and 10-month-old mice, as described previously [24].

### 2.4. Dissections

The dissection procedure was previously described [25]. Livers were weighed and divided for different analysis; the caudate lobes were postfixed in 4% paraformaldehyde in 0.1 M phosphate-buffered saline, pH 7.4 (PBS) and then stored in 70% ethanol at 4 °C until embedding in paraffin, with 100 mg of each hepatic left lobe designated for Western blotting (WB) and 50 mg for LC-MS.

The left gastrocnemius and 300 mg of each epididymal white AT (eWAT) were dissected for WB. All dissected tissues were frozen on dry ice immediately after dissection and stored at −80 °C until homogenization in lysis buffer.

### 2.5. Determination of Hormonal and Biochemical Parameters in Fasting Plasma

Blood glucose levels were measured using a glucometer (Arkray, Tokyo, Japan). In blood plasma, we further determined: insulin using an RIA kit (Merck Millipore, Burlington, MA, USA), leptin and fibroblast growth factor 21 (FGF21) using ELISA kits (both Merck Millipore, Burlington, NJ, USA), cholesterol and triacylglycerol using enzymatic photometric assays (Erba, Mannheim, Germany), and C-reactive protein (CRP) using a mouse CRP ELISA kit (Thermo Scientific, Frederick, MD, USA). All measurements were performed by following the manufacturer’s instructions.

### 2.6. Western Blotting

The WB protocol was described previously [26]. The appropriate dilutions of antibodies are provided in Table S1. The band intensities were normalized to the intensity of the band for either β-actin or Glyceraldehyde-3-phosphate Dehydrogenase (GAPDH) as the internal loading controls. To compare APP/PS1 and WT mice at all ages, samples of 3-month-old WT mice on HFD were applied to every gel, and the level was assessed as a baseline (100%).

### 2.7. Brain Immunohistochemistry

A detailed description of the chromogenic staining protocol is described in our previous study [26]. The dilution of primary antibodies is shown in Table S2.

For fluorescent immunohistochemistry, the free-floating sections were blocked in M.O. M (Vector Laboratories, Inc., Burlingame, CA, USA) to prevent nonspecific antibody binding (60 min at RT) and incubated with the primary antibody for 2 days at 4 °C; the appropriate dilutions are provided in Table S2. The sections were further incubated with goat anti-mouse IgG2b heavy chain (Biotin) (Abcam, Cambridge, Great Britain) at RT for 60 min, then with streptavidin Alexa Fluor™ 568 conjugate (Invitrogen/Thermo Fisher Scientific, Waltham, MA, USA) at RT for 60 min. Finally, the sections were incubated with NeuroTrace™ 435/455 Blue Fluorescent Nissl Stain (Invitrogen/Thermo Fisher Scientific, Waltham, MA, USA) to visualize the neurons.

The fibrillar Aβ plaques were stained using 1% thioflavin S (Merck Millipore, Burlington, MA, USA; incubation at RT for 30 min), followed by washing for 3 min in 80% ethanol, 90% ethanol and tap water and incubation with Autofluorescence Eliminator Reagent (Merck Millipore, Burlington, MA, USA).

For Aβ, Iba1, GFAP, phosphorylated Tau, total Tau and Neuronal Nuclear protein (NeuN) staining, images of the whole area of interest were taken at 10× magnification (8–10 sections per staining per mouse). The percentage of the area stained, selected manually according to the Paxinos and Franklin mouse brain atlas [27], was analyzed using ImageJ software (NIH, Bethesda, MD, USA). The threshold was kept constant for all samples in each staining experiment. For doublecortin (DCX) and Tau-3R staining, the numbers of DCX+ and Tau 3R+ cells, respectively, were counted manually using the ImageJ Multi-Point tool. The results are expressed as a percentage of the control group marked in every study to enable comparison of different staining series.

### 2.8. Brain MALDI MSI

Preparation of brain free-floating sections (*n* = 3) for mass spectrometry imaging (MSI) analysis and 1,5-diaminonaphthalene (DAN) matrix spraying was performed according to our previously published method [28]. SCiLS Lab 2016b software (SCiLS GmbH, Germany) was used for statistical analysis as described previously [29].

### 2.9. Liver Histology

Steatosis was examined using hematoxylin/eosin staining as described previously [30]. For fibrotic liver staining, the liver samples were deparaffinized and the rehydrated slides were transferred into Weigert’s hematoxylin solution for 10 min and rinsed for 5 min in tap water to remove the rest of the solution. The washed samples were stained with Picrosirius red solution (0.25 g of Sirius Red, 250 mL of 1.3% Picric acid solution) for 15 min. Next, the slices were dehydrated 3 times for 5 min in 99.99% ethanol and then 2 times for 2 min in xylene and mounted with DPX medium (Sigma-Aldrich, St. Louis, MO, USA). Histological images were obtained at 20× magnification under an Olympus IX83 inverted microscope (Olympus, Tokyo, Japan). The level of fibrosis was scored according to Kleiner’s study [31].

### 2.10. LC-MS

Lipids from liver (wet weight 50 mg, *n* = 5) and frontal cortex tissue (wet weight 10 mg, *n* = 5) were extracted using the BUME extraction method [32]. Untargeted lipidomics profiling was performed using the UPLC/ESI-MS method. The instrument was hyphenated from an UltiMate 3000 ultrahigh-performance liquid chromatography system and an Orbitrap Fusion Lumos Tribrid mass spectrometer (Thermo Fisher Scientific, Waltham, MA, USA) with heated electrospray ionization. Mobile phase A was 60:40 (*v*:*v*) acetonitrile/water, and mobile phase B was 90:10 (*v*:*v*) IPA/acetonitrile. Both contained 10 mM ammonium formate and 0.1% formic acid. A Waters Acquity UPLC BEH C18 (2.1 × 50 mm, 1.7 μm) column was operated at 45 °C and a flow rate of 180 μL/min. The injection volume was 5 μL for the positive mode and 10 μL for the negative mode. Full scan and MS/MS data acquisitions were obtained in the data-dependent analysis mode for both ionization modes separately in the range of 250–1200 Da. An MS resolution of 120,000 and MS/MS resolution of 15,000 were applied. The electrospray ionization voltage was set to 3.5 kV, and the transfer capillary was set to 320 °C. The identification of lipids was performed using LipidSearch 4.2 (Thermo Fisher Scientific, Waltham, MA, USA) based on precursor and product ions. The lipid intensities were normalized via internal standard normalization using the SPLASH™ Lipidomix^®^ standard (Avanti Polar Lipids, Alabaster, AL, USA). Liver lipids were only analyzed in 6-month-old APP/PS1 mice and WT mice on HFD as at this age, the mice developed the most significant steatosis. Mice on STD were not analyzed as we wanted to compare the effect of the diet on different mouse genotypes. Lipids in the frontal cortex were analyzed only in 10-month-old mice as in this age HFD worsened the brain pathology the most.

### 2.11. NMR-Based Metabolomics of Urine, Plasma, and Liver Samples

NMR-based metabolomics of urine, plasma and liver were performed using the protocol described by Pelantová et al. [33]. The individual metabolites were identified using Chenomx software (Chenomx Inc., Edmonton, AB, Canada) and by comparison with the HMDB database, [34]) or previously published data.

### 2.12. Statistical Analyses

The data are presented as the means ± SDs. The metabolic parameters and data from histology, immunohistochemistry and WB were tested for normality using the Shapiro–Wilk test and subsequently analyzed using one-way ANOVA with the Bonferroni post hoc test to allow comparisons among all mouse groups. The statistical analyses were performed with GraphPad Prism Software (San Diego, CA, USA), and *p* < 0.05 was considered statistically significant.

The rate of insulin resistance is expressed as the homeostatic model assessment index (HOMA-IR), calculated as (fasting glucose level, mmol/L) × (fasting insulin level, pmol/L) divided by 22.5 [35].

An untargeted multivariate analysis of the NMR data, based on the analysis of equidistantly binned (bin width = 0.01 ppm) and Pareto scaled spectra, was performed in MetaboAnalyst 4.0 software [36]. In the next step, based on the Lilliefors test for normality, the significance of changes induced by HFD and genetic background was evaluated using parametric one-way ANOVA on a set of all quantifiable signals in the spectra of urine, plasma and liver extracts.

A statistical analysis of the LC–MS data was performed using MetaboAnalyst 4.0 software. Before statistical analysis, the data were subjected to log transformation and Pareto scaling. A volcano plot analysis was used to identify altered lipids between models.

## 3. Results

### 3.1. Effect of HFD on Body Weight, at Weight and Lipid Profile

**Figure 2:**
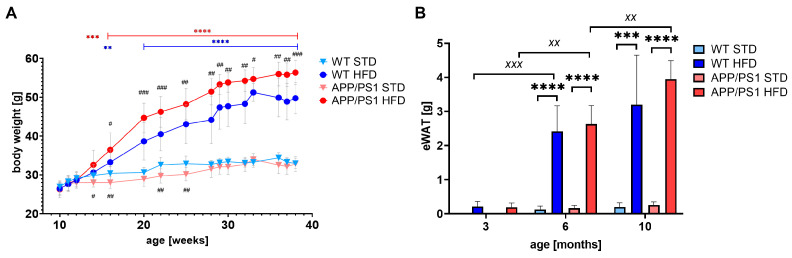
HFD significantly increased BW and eWAT weight. Body weights (**A**) and eWAT weights (**B**) in the APP/PS1 mice and their age-matched controls on STD or HFD at 3, 6, or 10 months of age. The data are presented as the means ± SD. Statistical analysis was made by (**A**) mixed-effects analysis with Dunnett post hoc test and (**B**) one-way ANOVA with Bonferroni post hoc test (*n* = 5–12 mice per group). The significances of changes induced by diet: **: *p* < 0.01, ***: *p* < 0.001 and ****: *p* < 0.0001; by genotype: #: *p* < 0.05, ##: *p* < 0.01, ###: *p* < 0.001; by age: ^xx^: *p* < 0.01, ^xxx^: *p* < 0.001.

As shown in Figure 2A, both APP/PS1 and WT mice fed HFD developed severe obesity compared to their age-matched controls on STD that did not significantly gain weight throughout the whole experiment. Even though the APP/PS1 STD group was leaner than the WT STD group, the APP/PS1 HFD group showed significantly higher BW from the sixth week on HFD (age 3.5 months) than the APP/PS1 STD group, while the WT HFD group showed significantly higher BW only from the eighth week on HFD (age 4 months) when compared to the WT STD group. The significant increase in BW of APP/PS1 mice on HFD in comparison with WT on HFD appeared at the age of 5 months. Accordingly, the eWAT weights were significantly increased by the HFD at 6 and 10 months of age in both mouse genotypes (Figure 2B).

Along with AT weight, the HFD significantly increased the plasma level of leptin in all 6- and 10-month-old mice (Table 1). A similar trend was observed for plasma cholesterol and plasma triacylglycerols (TGs). The fasting glucose levels were increased above the normoglycemic value (the value for WT mice) in mice on a HFD at all ages but were significant only at the age of 6 months. Obesity was also accompanied by hyperinsulinemia of both genotypes at the ages of 6 and 10 months; in addition, 10-month-old APP/PS1 mice on a HFD reached insulin levels that were 38-fold higher than those of their controls on a STD and 3-fold higher than those of age-matched WT controls on a HFD (Table 1).

### 3.2. HFD Caused Glucose Intolerance in 6-Month-Old Animals and Peripheral Insulin Resistance Increasing with Aging

All mice fed a HFD showed a significantly higher (HOMA-IR) index than their age- and genotype-matched STD-fed mice (Table 1), which indicates peripheral IR. The HOMA-IR of 10-month-old APP/PS1 mice on a HFD was also significantly increased in comparison with their age-matched WT controls on a HFD. Moreover, 10-month-old APP/PS1 mice on a HFD had significantly increased levels of fibroblast growth factor 21 (FGF21) in comparison not only with their age-matched APP/PS1 on a STD but also with their age-matched WT mice on a HFD.

**Figure 3:**
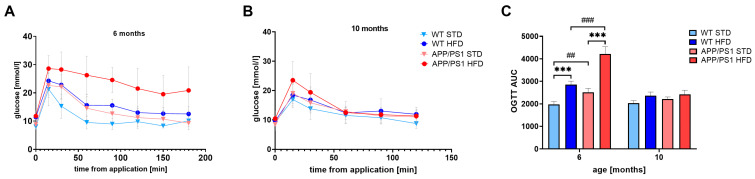
Oral glucose tolerance test in 6- and 10-month-old mice on STD and HFD. Time course of OGTT after orally administered glucose at a dose 2 g/kg at 6 months (**A**) and 10 months (**B**) of age and area under curve (AUC) (**C**). The data are presented as the means ± SD. Statistical analysis was made by one-way ANOVA with Bonferroni post hoc test (*n* = 5–12 mice per group). The significance of changes induced by diet: ***: *p* < 0.001, by genotype: ##: *p* < 0.01, ###: *p* < 0.001.

At the ages of 6 and 10 months, oral glucose tolerance tests (OGTTs) were performed. The HFD significantly increased the area under the curve (AUC) in 6-month-old mice of both genotypes, suggesting glucose intolerance (Figure 3). In addition, APP/PS1 mice on a HFD showed significantly increased glucose intolerance in comparison with HFD-fed WT controls, and APP/PS1 mice on a STD were glucose-intolerant in comparison with STD-fed WT controls. However, at 10 months of age, no differences were apparent among the groups.

### 3.3. HFD Caused Peripheral Inflammation in APP/PS1 Mice

**Figure 4:**
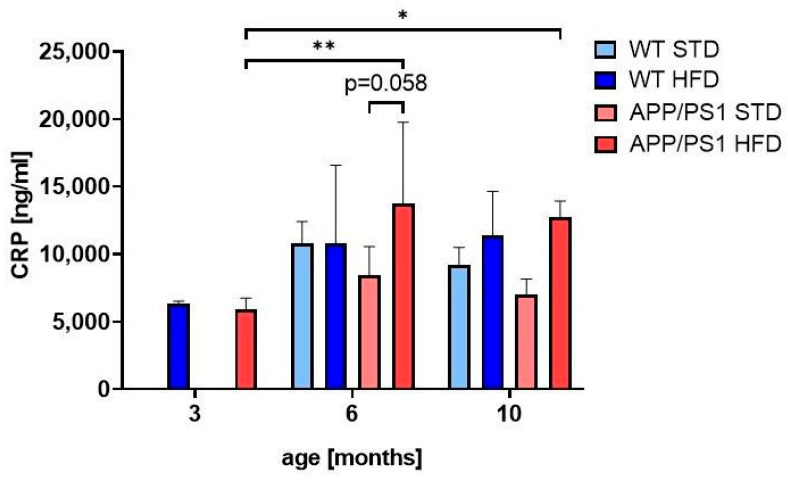
HFD caused peripheral inflammation in APP/PS1 mice. Quantification of CRP in fasted blood plasma. The data are presented as the means ± SD. Statistical analysis was made via one-way ANOVA with Bonferroni post hoc test (*n* = 5–7 mice per group). The significance of changes induced by age: *: *p* < 0.05, **: *p* < 0.01.

HFD-induced obesity was related with growing age to a significant increase in CRP, a marker of peripheral inflammation, only in the fasted plasma of APP/PS1 mice at 6 and 10 months of age (Figure 4) in comparison with 3-month old mice on a HFD.

### 3.4. HFD Caused Liver Steatosis in APP/PS1 Mice and Worsened Fibrosis in 10-Month-Old Animals

**Figure 5:**
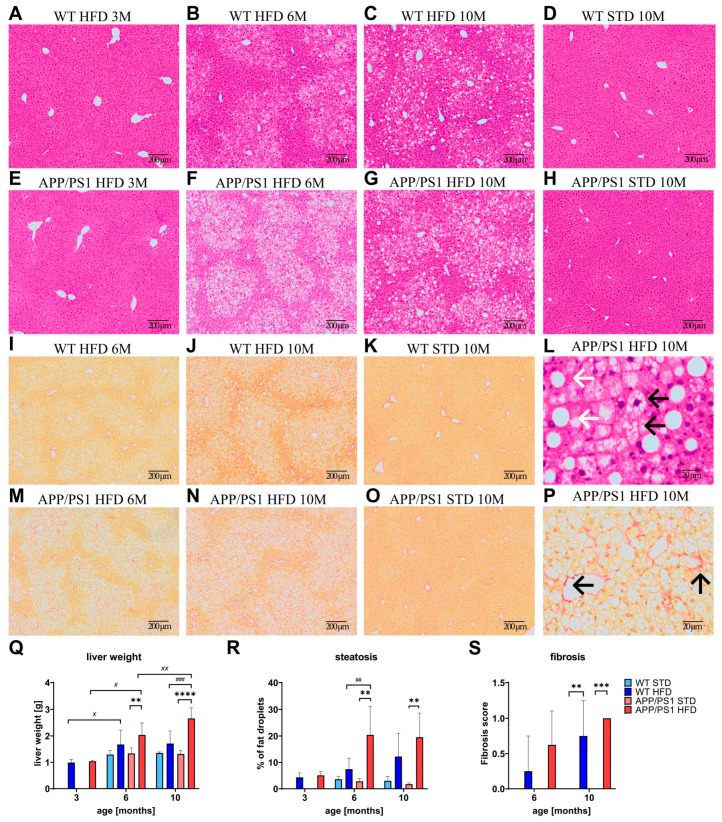
HFD caused liver steatosis and fibrosis. Quantification of liver weights (**Q**) and representative photomicrographs of the APP/PS1 mice on HFD and their controls on STD. Livers stained by hematoxylin-eosin for (**A**–**H**) steatosis and by pico-sirius red solution for fibrosis (**I**–**K**,**M**–**O**), and their quantification (**R**,**S**). Detailed photomicrographs of 10-month-old APP/PS1 mouse on HFD (**L**,**P**). White arrows in (**L**) show macrosteatosis and black arrows show microsteatosis of the liver. Black arrows in (**P**) point to fibrotic scarring. The data are presented as the means ± SD. Statistical analysis was made via one-way ANOVA with Bonferroni post hoc test (*n* = 4–10 mice per group). The significance of changes induced by diet: **: *p* < 0.01, ***: *p* < 0.001, ****: *p* < 0.0001, by age: ^X^: *p* < 0.05, ^XX^: *p* < 0.01, by genotype: ##: *p* < 0.01, ###: *p* < 0.001.

HFD feeding caused a significant increase in liver weight in 6- and 10-month-old APP/PS1 mice, while it only tended to increase the liver weight of WT mice (Figure 5Q). Furthermore, the livers of 10-month-old APP/PS1 mice on a HFD were significantly heavier than those of their respective WT controls on a HFD. The increase in liver weight was mainly caused by abnormal retention of fat within the liver and obvious liver steatosis. Representative images of steatotic liver slices are shown in Figure 5A–H. At the ages of 6 and 10 months, both APP/PS1 and WT mice on a HFD developed micro- and macrosteatosis (Figure 5L). Macrosteatosis is characterized by a single, bulky fat vacuole in the hepatocyte, displacing the nucleus to the edge of the cell. In microsteatosis, the cytoplasm of the hepatocytes contains tiny lipid vesicles without nuclear dislocation. Only in APP/PS1 mice fed a HFD was the increase in steatosis statistically significant. Furthermore, APP/PS1 mice fed a HFD developed a significant gain in steatosis at 6 months compared to WT mice fed a HFD (Figure 5R). STD-fed mice of both genotypes did not show any evidence of liver steatosis, even at the age of 10 months (Figure 5D,H).

The level of fibrosis stained with Picrosirius red solution was measured only in 6- and 10-month-old mice after they developed steatosis. Representative images of fibrotic liver slices are shown in Figure 5I–K,M–O. APP/PS1 and WT mice on a HFD developed mild fibrosis at 6 months of age compared to their age-matched controls on a STD (Figure 5P), which was significantly pronounced in both strains at 10 months of age (Figure 5S). Mice on a STD did not develop any fibrosis.

### 3.5. HFD Altered Metabolic Profiles of Urine, Plasma and Polar Liver Extracts in APP/PS1 Mice

**Figure 6:**
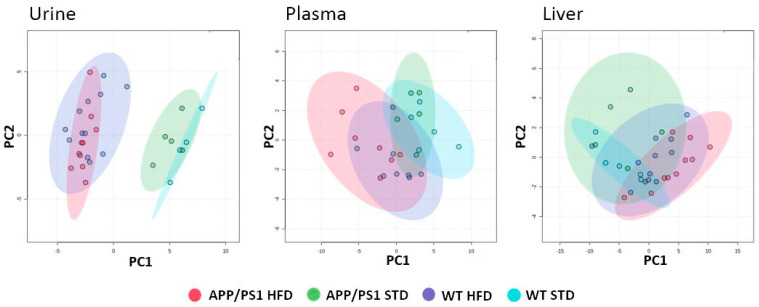
HFD altered urinary, plasma and liver metabolic profiles in APP/PS1 mice. PCA score plots based on 1H NMR spectra of urine, plasma, and polar lipid extract samples collected at 6 (plasma) or 10 (urine, liver) months of age. N = 5 in STD groups (except for 4 urine samples in WT STD) and 8–12 in HFD groups.

Metabolic profiles of APP/PS1 and WT mice were analyzed in urine and plasma at 6 and 10 months of age and in polar liver extracts at 10 months of age using NMR-based metabolomics. An untargeted urine analysis showed that the changes between groups were driven solely by the effect of diet, not strain (Figure 6). The PCA model of plasma and liver samples displayed only a trend in the separation of APP/PS1 mice on the HFD and STD.

A univariate analysis of 71 signals in urine showed that the HFD significantly altered very similar sets of metabolites at 6 and 10 months old in both WT and APP/PS1 mice (Supplementary Tables S3 and S4). These changes are typically observed in various rodent models of obesity, in particular an increase in the levels of 1-methylnicotinamide, N-carbamoyl-β-alanine, choline, glycine and creatinine and a decrease in the levels of hippurate, acylglycines, metabolites of the tricarboxylic acid cycle and oxoacids in obese compared to lean animals.

The effect of the HFD became apparent in the plasma at 6 months with an increase in the concentrations of glucose, arabinose, glycerol and dimethylglycine and a decrease in the concentrations of several amino acids and hydroxyacids (Supplementary Table S5). At 10 months of age, the difference between strains was reflected in the HFD-fed animals by an increase in lactate and alanine levels and a decrease in 3-hydroxybutyrate in APP/PS1 mice compared with WT mice (Supplementary Table S6).

The HFD had a significant effect on the distribution of polar metabolites only in the livers of APP/PS1 mice but not in WT controls. In particular, an increase in the concentrations of saccharides, glycero-phosphocholine and lactate and a decrease in the concentrations of nucleic bases, several amino acids, taurine and glycerol in obese compared to lean APP/PS1 mice were detected (Supplementary Tables S7 and S8).

### 3.6. HFD Upregulated Liver Lipid Diversity in 6-Month-Old APP/PS1 Mice

**Figure 7:**
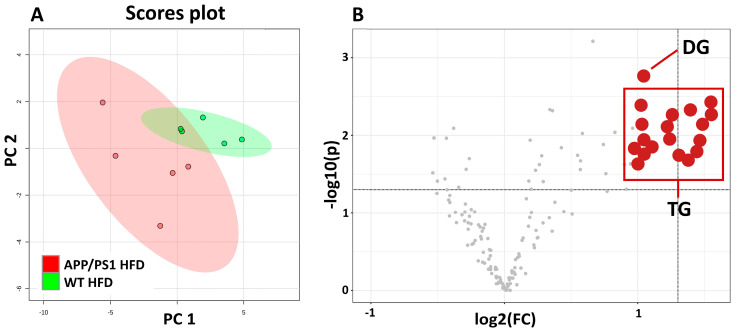
HFD upregulated liver lipids in 6-month-old APP/PS1 mice. PCA score plot (**A**) based on liver lipid profiling of WT and APP/PS1 (APP) mice on HF diets at age 6 months. Volcano plot of liver lipids (**B**) indicating lipid species that are significantly increased in APP/PS1 mice on HF diet. Statistical analysis was made between groups via one-way Student’s *t*-test (n = 5 mice per group). DG: diacylglycerol; TG: different species of triacylglycerols specified in Supplementary Table S9.

Untargeted lipidomic profiling of liver homogenates identified a total of 157 lipids. A PCA analysis (Figure 7A) based on lipid profiling revealed a separation between 6-month-old WT mice and APP/PS1 mice on a HFD. Higher diversity was found between APP/PS1 samples. A volcano plot using a significance level of *p*-value < 0.05 based on Student’s *t*-test and a fold change (FC) value higher than 2 (Figure 7B, Supplementary Table S9) revealed 19 upregulated lipids in APP/PS1 mice on the HFD in comparison to WT mice on the same diet. One species belonged to the diacylglycerols, and the other eighteen were represented by TGs. Furthermore, the majority of these altered glycerolipids mostly contained saturated fatty acids (16:0, 18:0) and monounsaturated fatty acids (16:1, 18:1).

### 3.7. HFD and Age Attenuated PI3K/Akt Signaling in the Liver, eWAT and Skeletal Muscle

**Figure 8:**
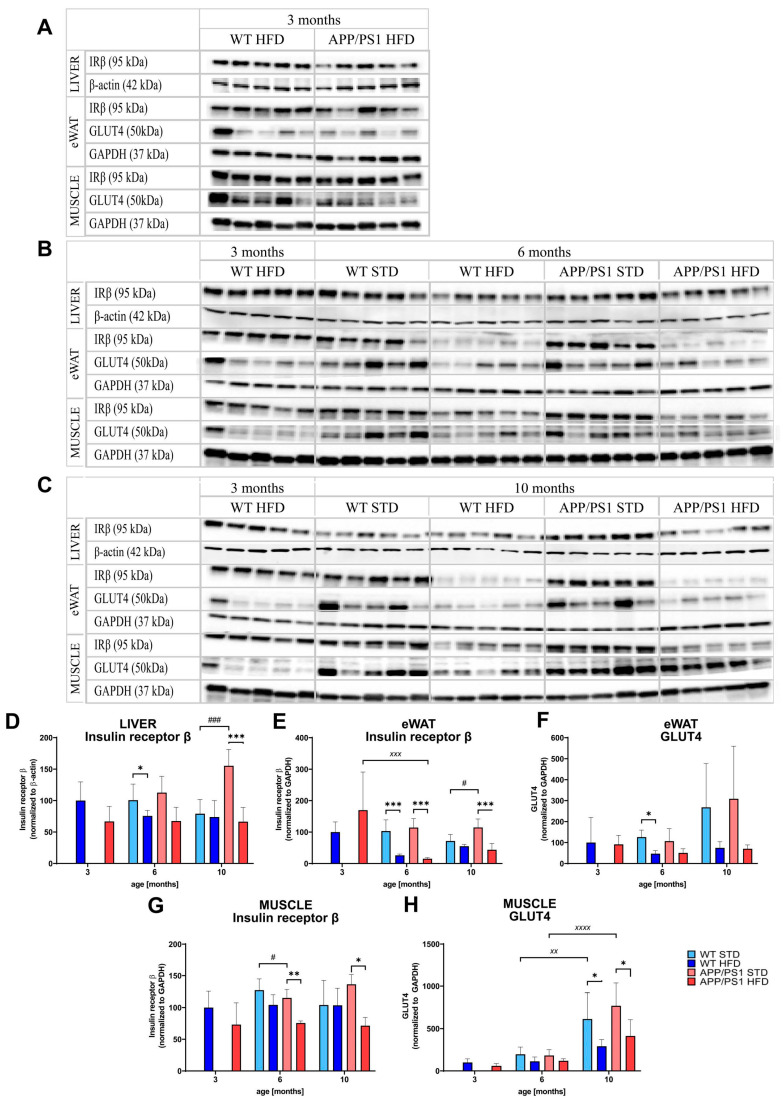
HFD and age attenuated PI3K/Akt signaling in the liver, eWAT and skeletal muscle. Proteins levels were determined via Western blotting. Immunoblots at 3 months (**A**), 6 months (**B**) and 10 months (**C**) of age, quantification of protein levels (**D**–**H**): insulin receptor β in the liver, (**D**) insulin receptor β (**E**) and GLUT4 (**F**) in eWAT, insulin receptor β (**G**) and GLUT4 (**H**) in muscle. Percentage of the stained area is expressed as a percentage of 3-month-old WT on HFD to enable the comparison of multiple staining series. The data are presented as the means ± SD. Statistical analysis was made via one-way ANOVA with Bonferroni post hoc test (*n* = 5 mice per group). The significance of changes induced by diet: *: *p* < 0.05, **: *p* < 0.01, ***: *p* < 0.001; by age: ^XX^: *p* < 0.01, ^XXX^: *p* < 0.001, ^XXXX^: *p* < 0.0001, by genotype: #: *p* < 0.05, ###: *p* < 0.001.

HFD consumption tended to decrease the level of insulin receptor β (IRβ) in the liver and skeletal muscle of 6-month-old WT mice fed the HFD and significantly decreased the level of IRβ in the eWAT. In the APP/PS1 mice, HFD feeding significantly reduced the level of IRβ at both ages in all mentioned peripheral tissues (Figure 8).

As shown in Table 2 and Supplementary Figure S1, phosphoinositide 3-kinase (PI3K), total Akt and p-Akt (Ser473) tended to decrease after HFD consumption in both genotypes at the ages of 6 and 10 months. However, we did not observe any consistent trend in the level of these kinases with aging, since PI3K and Akt tended to decrease with aging in the liver and skeletal muscle, while PI3K in the eWAT increased.

In the eWAT and skeletal muscle, GLUT4 levels increased with aging (Figure 8). The HFD significantly reduced the level of GLUT4 in the skeletal muscle of both WT and APP/PS1 10-month-old mice.

### 3.8. HFD Exacerbated Aβ Plaque Load in the Cortices of APP/PS1 Mice

**Figure 9:**
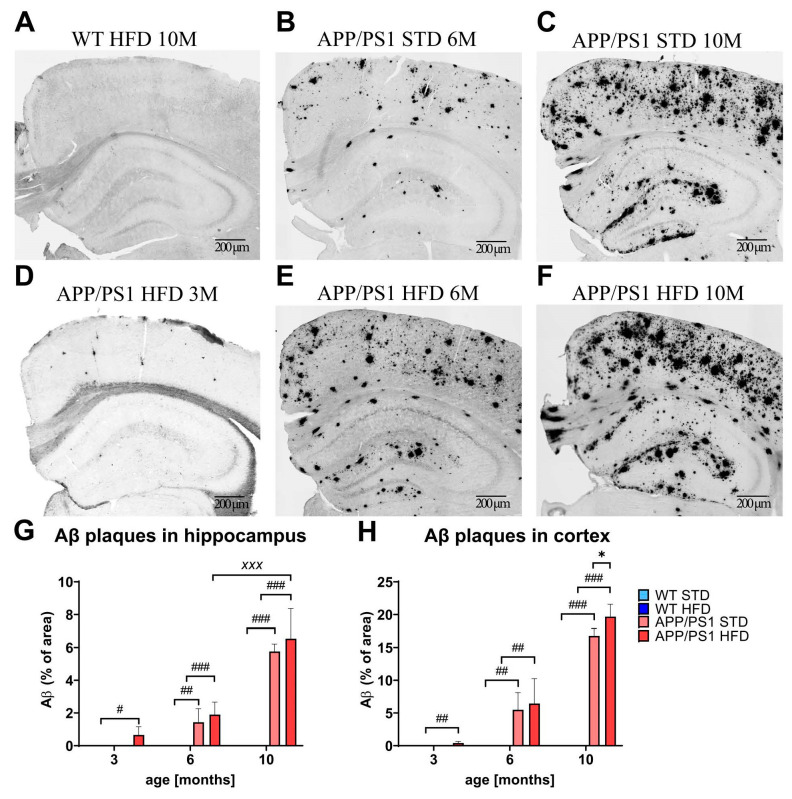
HFD exacerbated Aβ plaque load in the cortices of 10-month-old APP/PS1 mice. Representative photomicrographs of the brains of APP/PS1 mice fed either with STD at 6 months or (**B**) 10 months (**C**) or HFD at 3 months (**D**), 6 months (**E**) and 10 months (**F**) of age and the WT control at 10 months of age (**G**) immunohistochemically stained (**A**–**F**) for human Aβ and their quantification (**G**,**H**). Aβ plaque load is expressed as a percentage of the stained area. The data are presented as the means ± SD. Statistical analysis was made via one-way ANOVA with Bonferroni post hoc test (*n* = 5–8 mice per group). The significance of changes induced by diet: *: *p* < 0.05, by age: ^XXX^: *p* < 0.001, by genotype: #: *p* < 0.05, ##: *p* < 0.01, ###: *p* < 0.001.

Photomicrographs of immunohistochemically stained brain sections showed the development of extensive Aβ plaque loads in both the hippocampi and cortices of APP/PS1 mice (Figure 9), starting at 3 months and further developing with age. As expected, control mice did not develop any plaques, even at 10 months of age. HFD significantly exacerbated the Aβ plaque loads in the cortices of 10-month-old APP/PS1 mice (Figure 9F), compared to those of APP/PS1 mice on the STD.

### 3.9. HFD Worsened Neuroinflammation in the Brains of APP/PS1 Mice

**Figure 10:**
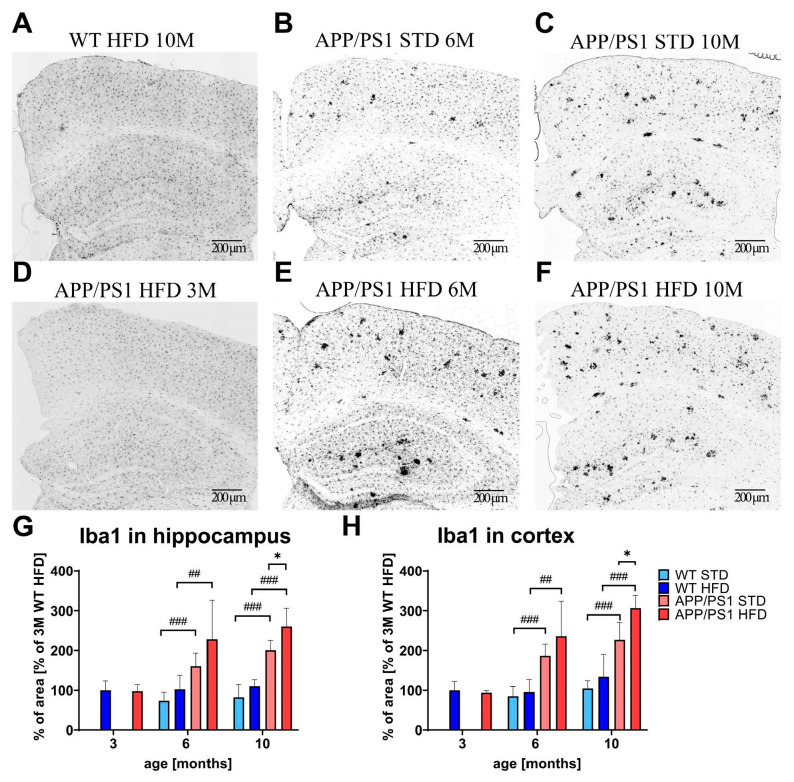
Effect of HFD on microgliosis in the hippocampi and cortices of the APP/PS1 mice. Representative photomicrographs of the brains of APP/PS1 mice fed either with STD at 6 months (**B**) or 10 months (**C**) or HFD at 3 months (**D**), 6 months (**E**) and 10 months (**F**) of age and the WT control at 10 months of age (**A**) immunohistochemically stained (**A**–**F**) and their quantification (**G**,**H**). Percentage of the stained area is expressed as a % of the 3-month-old WT mice on HFD to enable the comparison of multiple staining series. The data are presented as the means ± SD. Statistical analysis was made via one-way ANOVA with Bonferroni post hoc test (*n* = 5–8 mice per group). The significance of changes induced by diet: *: *p* < 0.05, by genotype: ##: *p* < 0.01, ###: *p* < 0.001.

Immunohistochemical microglial staining of Iba1 revealed visible clusters of activated microglia in the hippocampi and cortices of APP/PS1 mice on STD at both ages (Figure 10) but not in the brains of WT control mice, where only resting microglia were visible (Figure 10A). HFD feeding significantly increased the level of microgliosis in both the hippocampi and cortices of 10-month-old APP/PS1 mice on the HFD compared to APP/PS1 mice on the STD (Figure 10).

**Figure 11:**
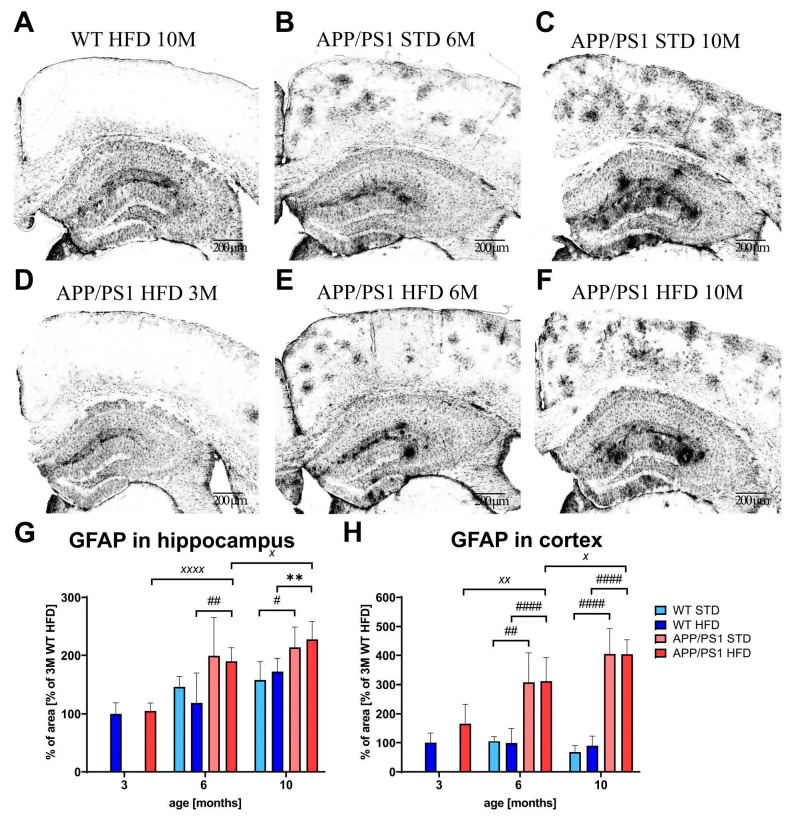
Effect of HFD on astrocytosis in the hippocampi and cortices of the APP/PS1 mice. Representative photomicrographs of the brains of APP/PS1 mice fed either with STD at (**B**) 6 months or (**C**) 10 months or HFD at (**D**) 3 months, (**E**) 6 months and (**F**) 10 months of age and (**A**) the WT control at 10 months of age, immunohistochemically stained (**A**–**F**) for GFAP, and their quantification (**G**,**H**). Percentage of the stained area is expressed as a % of the 3-month-old WT mice on HFD to enable the comparison of multiple staining series. The data are presented as the means ± SD. Statistical analysis was made via one-way ANOVA with Bonferroni post hoc test (*n* = 5–8 mice per group). The significance of changes induced by age: ^X^: *p* < 0.05, ^XX^: *p* < 0.01, ^XXXX^: *p* < 0.0001, by genotype: #: *p* < 0.05, ##: *p* < 0.01, ####: *p* < 0.0001, by diet: **: *p* < 0.01.

Immunohistochemical staining of the astrocytic marker GFAP revealed clusters of reactive astrocytes in the hippocampi and cortices of APP/PS1 mice on both diets (Figure 11); the HFD did not affect the number of reactive astrocytes at any age. However, astrogliosis significantly increased with age in both the hippocampi and cortices of APP/PS1 mice. Moreover, correlation was found between peripheral (CRP) and central (GFAP) neuroinflammation that was calculated not only in the whole set of mice (Pearson r: 0.3924, *p* = 0.0028 **) but also separately in WT mice (Pearson r: 0.4634, *p* = 0.0130 *).

### 3.10. HFD Increased Tau Phosphorylation around Aβ Plaques in the Hippocampi and Cortices of 6-Month-Old APP/PS1 Mice

**Figure 12:**
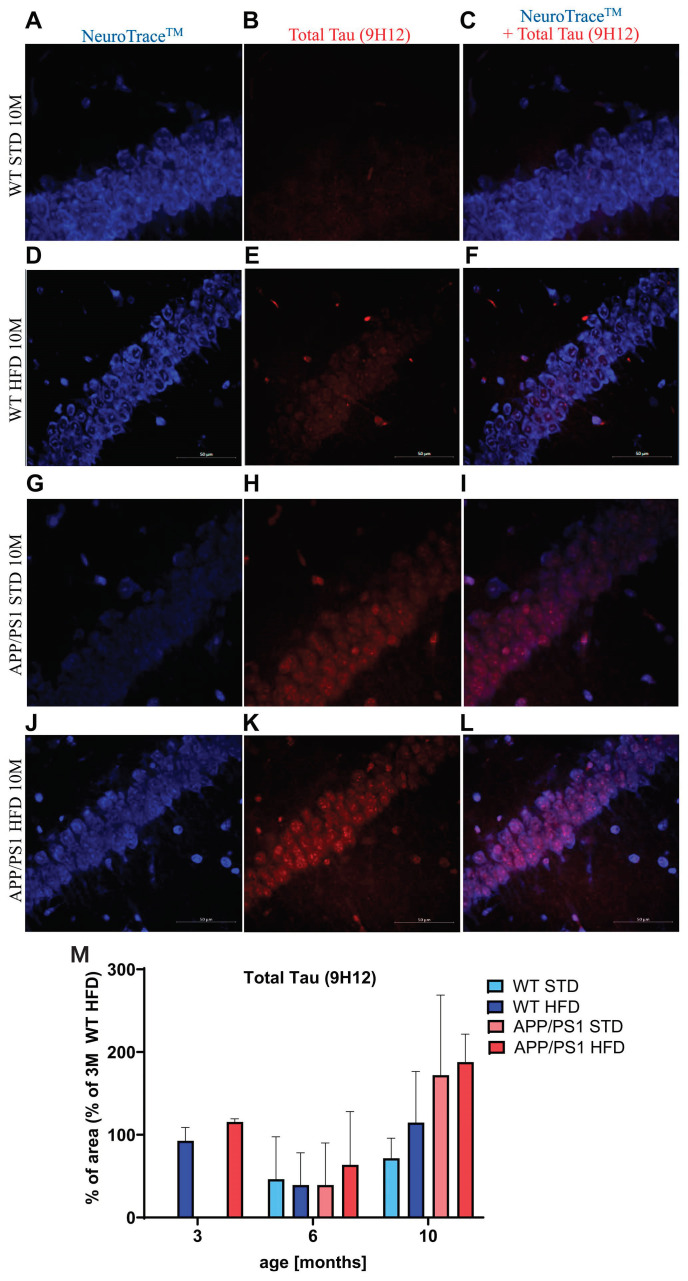
Accumulation of Tau in the CA1 part of hippocampi of the 10-month-old mice. Representative photomicrographs of the hippocampi of WT mice on STD (**A**–**C**), WT mice on HFD (**D**–**F**), APP/PS1 mice on STD (**G**–**I**) and APP/PS1 mice on HFD (**J**–**L**) with NeuroTraceTM (**A**,**D**,**G**,**J**), total Tau (9H12) (**B**,**E**,**H**,**K**) and their combination (**C**,**F**,**I**,**L**) presented. The quantification is shown in (**M**) and is expressed as a percentage of the 3-month-old WT mice on HFD to enable the comparison of multiple staining series. The data are presented as the means ± SD. Statistical analysis was made via one-way ANOVA with Bonferroni post hoc test (*n* = 5–8 mice per group).

Double staining of total Tau (antibody 9H12), which recognizes the central region of Tau protein (aa162-175), and NeuroTrace™ visualization of neurons revealed an increasing trend in the accumulation of Tau protein in the neurons of the cornu ammonis (CA1) part of the hippocampus in 10-month-old APP/PS1 mice on both diets compared to their age-matched WT controls (Figure 12); HFD did not affect the accumulation of Tau.

**Figure 13:**
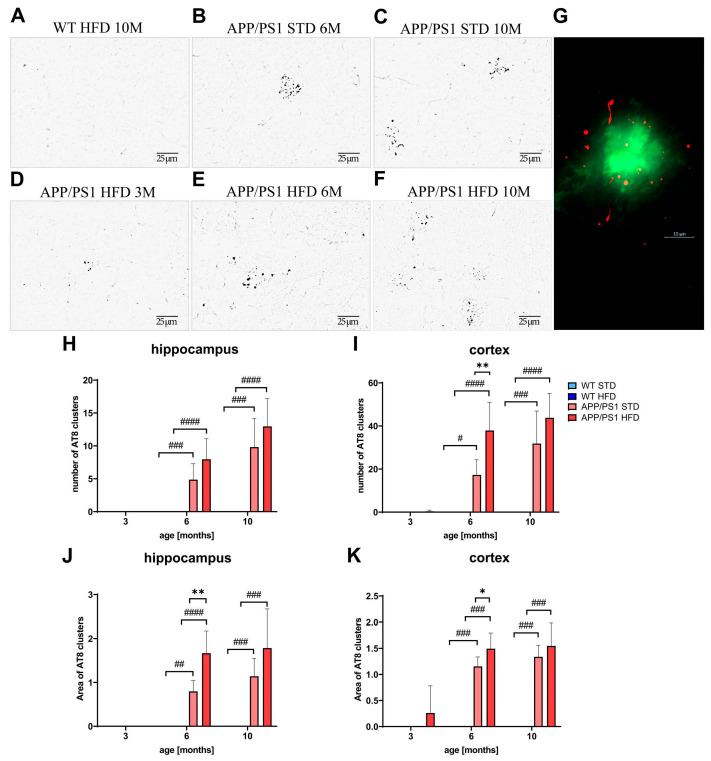
Increased Tau phosphorylation around Aβ plaques in hippocampi and cortices of APP/PS1 mice. Representative photomicrographs of the APP/PS1 mice fed either with STD at (**B**) 6 months or (**C**) 10 months or HFD at (**A**) 3 months, (**E**) 6 months and (**F**) 10 months of age and (**D**) the WT control at 10 months of age, immunohistochemically stained (**A**–**C**) with AT8 antibody recognizing p-Tau at Ser202 and Thr205, and their quantification (**H**–**K**). (**G**) Representative figure of double staining of Aβ plaque (Thioflavin S) and p-Tau (AT8 antibody) in 10-month-old APP/PS1 mouse. Percentage of the stained area is expressed as a percentage of the 3-month-old WT mice on HFD to enable the comparison of multiple staining series. The data are presented as the means ± SD. Statistical analysis was made via one-way ANOVA with Bonferroni post hoc test (*n* = 5–8 mice per group). The significance of changes induced by diet: *: *p* < 0.05, **: *p* < 0.01, by genotype: #: *p* < 0.05, ##: *p* < 0.01, ###: *p* < 0.001, ####: *p* < 0.0001.

Immunohistochemical staining revealed an increase in the amount of Tau phosphorylated at Ser202 and Thr205 (AT8 antibody) in dystrophic neurites (Figure 13) in the hippocampi and cortices of APP/PS1 mice. Tau phosphorylation was detectable in 3-month-old APP/PS1 mice (Figure 13D) and increased with age and the spread of Aβ pathology, as shown in Figure 13G, where pTau labeled with an anti-AT8 antibody (red color) was detectable around Aβ plaques labeled with thioflavin S (green color).

The HFD significantly increased the number of AT8 clusters formed around the Aβ plaques in the cortices and the size of the AT8 clusters in the hippocampi and cortices of 6-month-old APP/PS1 mice (Figure 13).

### 3.11. HFD and Age Attenuated the PI3K/Akt Signaling Pathway in the Hippocampus

**Figure 14:**
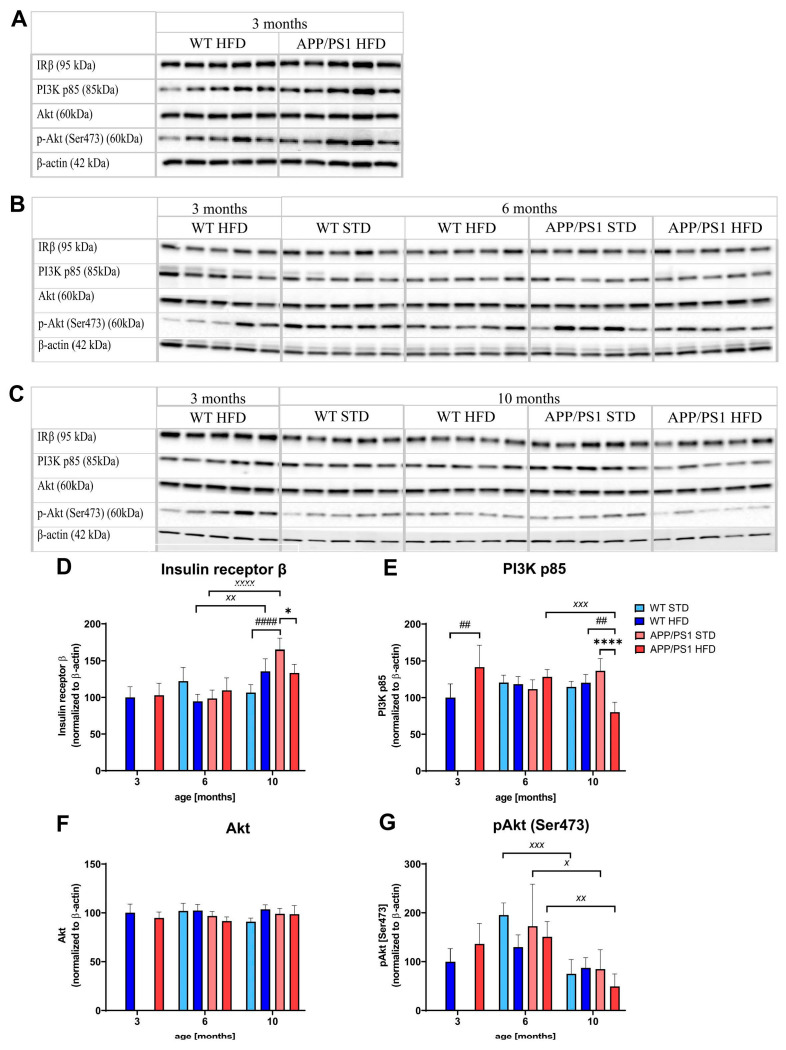
HFD and age attenuated the PI3K/Akt signaling pathway in the hippocampus. Proteins were determined using Western blotting. Immunoblots at (**A**) 3 months, (**B**) 6 months and (**C**) 10 months of age, (**D**–**G**) quantification of protein levels: (**D**) Insulin receptor β, (**E**) PI3K p85, (**F**) Akt, (**G**) p-Akt (Ser473). Percentage of the stained area is expressed as a % of 3-month-old WT on HFD to enable the comparison of multiple staining series. The data are presented as the means ± SD. Statistical analysis was made via one-way ANOVA with Bonferroni post hoc test (*n* = 5–8 mice per group). The significance of changes induced by diet: *: *p* < 0.05, ****: *p* < 0.0001, by age: ^X^: *p* < 0.05, ^XX^: *p* < 0.01, ^XXX^: *p* < 0.001, ^XXXX^: *p* < 0.0001, by genotype: ##: *p* < 0.01, ####: *p* < 0.0001.

The level of IRβ significantly increased with age in the hippocampi of WT and APP/PS1 mice; however, HFD consumption reversed this effect. Significantly decreased IRβ was observed in the hippocampus of 10-month-old APP/PS1 mice fed a HFD compared to APP/PS1 mice fed a STD (Figure 14). Furthermore, the levels of PI3K p85 also significantly decreased in 10-month-old APP/PS1 mice on a HFD compared to their age-matched controls on a STD but also compared to 10-month-old WT mice on a HFD. No differences were observed in the levels of Akt; nevertheless, its phosphorylation at Ser473 tended to decrease with HFD consumption in 6-month-old WT and APP/PS1 mice and significantly decreased between the sixth and tenth months of age (Figure 14). Strong negative correlations were computed between increasing peripheral IR (HOMA-IR index), decreased hippocampal level of PI3K (Pearson r: −0.3226, *p* = 0.0253) and p-Akt (Ser473) (Pearson r: −0.2941, *p* = 0.0448) as markers of central IR.

### 3.12. Decreased Neuronal Density and Neurogenesis with Age of Mice

Immunohistochemical staining of NeuN, a marker of mature neurons, detected a significant decrease in neuronal density in the hippocampi of WT and APP/PS1 mice fed a HFD and in the cortices of WT mice fed a HFD between the third and sixth months of age (Supplementary Figure S2) and in the cortices of WT mice fed a HFD.

Immunohistochemical staining of DCX (Supplementary Figure S3), a marker of early neurogenesis, revealed a significant, sharp decrease in the production of new neurons in the dentate gyrus (DG) of APP/PS1 and WT mice between the ages of 3 and 6 months on a STD or HFD (Supplementary Figure S3A,B), which further decreased between the sixth and tenth months (Supplementary Figure S3C). Similarly, staining of Tau 3R, a marker of late neurogenesis, revealed a significantly decreased number of newly born neurons in the DG between the third and sixth months of age (Supplementary Figure S3D,E) in APP/PS1 and WT mice (Supplementary Figure S3F).

### 3.13. HFD Decreased Synaptogenesis in the Hippocampi of APP/PS1 Mice

The presynaptic marker synaptophysin tended to decrease in 6-month-old APP/PS1 mice compared to WT mice (Supplementary Figure S4B) but was significantly decreased in 10-month-old APP/PS1 mice both on a STD or HFD (Supplementary Figure S4C) compared to their diet-matched WT controls (Supplementary Figure S4D). Compared to APP/PS1 mice on the STD, the HFD further tended to decrease the level of synaptophysin in 6-month-old APP/PS1 mice but also tended to decrease the level of the postsynaptic marker spinophilin in 6-month-old APP/PS1 mice (Supplementary Figure S4E). The level of spinophilin also tended to decrease in 3-month-old APP/PS1 mice compared to the WT mice on a HFD (Supplementary Figure S4A). All results from the measured AD-like pathology parameters affected by age, HFD and APP/PS1 genotype are summarized in Table 3.

### 3.14. HFD Upregulated TGs in the Frontal Cortices of APP/PS1 Mice

From untargeted lipidomic profiling of the frontal cortex, a total of 127 lipids were identified. PCA analysis (Supplementary Figure S5) based on lipid profiling revealed no clear separation between WT mice and APP/PS1 mice on a HFD and STD at 10 months of age. The volcano plot revealed three upregulated TGs in APP/PS1 mice on the HFD in comparison to the STD (Supplementary Figure S6A). When comparing the frontal cortex lipidome of APP/PS1 mice and WT mice on the HFD, upregulation of lysophosphatidylethanolamine (18:0) was found (Supplementary Figure S6B). No more changes were found in the lipidome, probably because the results represent the average concentration values for the entire analyzed part of the tissue, and all spatial information about specific lipidome changes in the small regions was lost due to the tissue homogenization step during sample preparation. MSI brain analysis in negative ion mode revealed upregulation of membrane lipids, such as phosphatidylinositols and gangliosides, together with depletion of sulfatides. The most significant differences between 10-month-old APP/PS1 mice on a HFD compared to APP/PS1 on STD (Supplementary Figure S7) were found in the polar glycolipids-gangliosides (GM2 36:1 and GM3 36:1), which were previously found to be colocalized with senile plaques and astrocytosis [29,37]. However, plaque-like accumulation looked visually similar between the STD and HFD groups, and ROC analysis showed no significant difference.

## 4. Discussion

Our study aimed to determine a potential relationship between obesity-induced IR, inflammation in the periphery and AD-like pathology in the brains of APP/PS1 mice fed a HFD.

A growing body of evidence supports the idea that obesity, T2DM and AD share common pathological changes, such as low-grade chronic inflammation that further accentuates already present peripheral IR [38]. HFD-induced obesity may lead to IR and cause inflammation not only in the periphery but also in the brain. In concordance with studies of others [14,15,19], more pronounced HFD-induced obesity was observed in APP/PS1 mice, followed by increased amounts of the eWAT, resulting in elevated levels of plasma leptin, cholesterol, and TGs in 6- and 10-month-old mice.

Fasting blood glucose, as well as glucose concentration in urine, were significantly increased in 6-month-old HFD-fed mice independently of genotype, but these mice became only prediabetic, since their glucose levels did not reach the indicative level for diabetes [39,40,41]. However, glucose intolerance in OGTT was increased in 6-month-old APP/PS1 mice on STD compared to WT on STD, and was even more pronounced in APP/PS1 fed HFD, similarly to the study of Lee [16]. Plasma levels of insulin and HOMA-IR index were significantly increased in HFD-fed mice at both 6 and 10 months, and a further increase was observed in 10-month-old APP/PS1 mice, similarly to the studies of others [16,40]. Plasma concentration of FGF21, an adipokine whose concentration is increased in patients with obesity, was increased in 10-month-old animals; the increased concentration of plasma FGF21 in mice of both genotypes fed with a HFD at the age of 10 months could compensate for insulin resistance and finally increase tolerance to glucose through increased glucose uptake by insulin-sensitive tissues, which could be partly demonstrated by increased protein levels of GLUT4 in muscle and eWAT [42]. Obesity is associated with mild chronic inflammation, characterized by increased levels of CRP, which is further linked to the development of IR [43,44]. In our study, HFD-induced obesity promoted a significant increase in CRP only in APP/PS1 mice at both 6 and 10 months of age.

A metabolomic analysis revealed that the urinary metabolic profile was significantly affected by a HFD but not by the genetic background of the mice. Increases in 1-nicotinamide, its metabolite 2-PY and N-carbamoyl β-alanine in obese animals have previously been suggested as a suitable marker of obesity or diabetes [45,46]; similarly, decreases in acylglycines or hippurate have been repeatedly published in association with obesity [46,47,48].

The livers of both APP/PS1 and WT mice fed a HFD exhibited severe hepatomegaly beginning at the age of 6 months. Ten-month-old APP/PS1 mice had significantly higher liver weight than their WT controls. Furthermore, 6-month-old APP/PS1 mice developed significantly pronounced liver steatosis and started to develop fibrosis, unlike WT mice on the HFD, which developed mild steatosis and fibrosis only at the age of 10 months. These findings are supported by untargeted lipidomics, which showed accumulation of saturated and low-unsaturated TGs in the livers of APP/PS1 mice on HFD. In the livers of APP/PS1 mice fed HFD, metabolomic analysis revealed a significant increase in lactate, whose accumulation has been associated with steatosis [49], and a decrease in the glucogenic amino acids glycine and aspartate, which may reflect reduced gluconeogenesis induced by liver injury [49], as observed in rats fed with a HFD [50]. These results support increasing evidence of the involvement of fatty liver disease in aggravating AD pathogenesis, as the liver is considered a major player in the clearance of Aβ at the periphery [51,52,53].

Skeletal muscle, the liver and eWAT are major insulin-responsive tissues that control glucose homeostasis [54]. Glucose uptake in the muscle and AT is based on the insulin-dependent glucose transporter GLUT4, whose malfunction is known to be involved in obesity-induced IR [55,56]. Similarly to the studies of others [57,58,59], the HFD significantly decreased the level of muscular and adipose GLUT4 protein in both APP/PS1 and WT mice, suggesting a reduction in glucose uptake. In patients with obesity, decreased levels of insulin receptors are inversely related to the degree of hyperinsulinemia [54]. The HFD reduced the level of IRβ in the eWAT of both APP/PS1 and WT mice and in the livers and muscles of APP/PS1 mice. The levels of PI3K in the muscles and eWAT were strongly decreased, similarly to previous studies [54,60,61]. The HFD caused a decrease in the activation of Akt in the liver and eWAT, further confirming the impairment of the peripheral PI3K/Akt signaling pathway [62,63]. Impaired insulin signaling in peripheral tissues, caused by HFD consumption that was even more pronounced in APP/PS1 mice, could lead to obesity and T2DM as a result of IR, as described in the study of Huang [54].

The burden of senile plaques and the following astrocytic and microglial reactivity as well as Tau hyperphosphorylation were examined to demonstrate worsening of AD-like pathology and related neuroinflammation after long-term consumption of HFD. Six-month-old APP/PS1 mice fed HFD did not differ in the development of Aβ pathology from STD-fed ones, which confirms the studies of [64,65]. However, consistently with other studies [40], we observed a significant increase in Aβ plaque load in the cortices of 10-month-old APP/PS1 HFD mice. Similarly to our previous study, APP/PS1 mice developed a number of reactive astrocytes and microglia in close proximity of Aβ plaques. Although HFD did not affect the level of astrocytosis, a higher number of reactive microglia was found in the hippocampi and cortices of HFD-fed APP/PS1 mice, as described previously by [40], which supports the role of neuroinflammation in this animal model. Furthermore, the correlation between peripheral and central inflammation provides evidence that inflammation is one of the common denominators of obesity and AD.

The level of total Tau tended to increase in 10-month-old APP/PS1 mice on both diets compared to WT controls. However, in contrast to the study on 3xTg-AD mice [66], no progressive increase in total Tau was found in HFD-fed mice. The amyloid cascade theory of AD etiology states that the accumulation of Aβ plaques accelerates the progression of Tau pathology [67]. In this study, Aβ plaques induced the accumulation of endogenous phosphorylated Tau within dystrophic neurites surrounding the plaques of APP/PS1 mice, similarly to the study of Chen [68]. By 6 months of age, HFD already significantly aggravated both the number and size of Tau clusters surrounding the plaques. Hyperphosphorylated Tau is considered to be responsible for the cytotoxic effects of Aβ [3,69,70,71], suggesting that increased Tau pathology could further support the neuroinflammatory effect of Aβ in APP/PS1 mice.

PI3K/Akt signaling is believed to be impaired in the brains of obese subjects with IR [9]. HFD decreased the levels of IRβ and PI3K p85 proteins in the hippocampi of 10-month-old APP/PS1 mice, suggesting the development of central IR, which negatively correlates with increased HOMA-IR, a marker of peripheral IR. Moreover, the activation of Akt protein decreased in an age-dependent manner, supporting the most important role of age in the development of neurodegenerative diseases such as AD [72]. These data show that obesity-induced IR, demonstrated as hyperinsulinemia, could lead to reduced activation of insulin signaling in the brain and provide evidence of IR being an interconnection between obesity and AD-like pathology development.

The loss of synapses and neurons has been correlated with memory impairment as an early event during AD progression [9]. Although several studies report impaired hippocampal neurogenesis in HFD-fed mice [73,74], no significant difference was found in either neuronal density or early and late neurogenesis of APP/PS1 and WT mice on a HFD, similarly to the study of Rupp [75]. However, our present study confirmed the previously reported decrease in synaptophysin in APP/PS1 mice [26]; moreover, a decrease in synaptic markers was further pronounced in HFD-fed mice, similarly to the studies of others [76,77].

## 5. Conclusions

In conclusion, HFD feeding in combination with mutations leading to the familial form of AD caused more profound metabolic disturbances, such as obesity, glucose intolerance, IR and chronic peripheral inflammation. These metabolic disturbances were followed by impaired hippocampal insulin signaling, leading to a worsening of Aβ and Tau pathology, as well as neuroinflammation in obese APP/PS1 mice. All together, our data support the theory of IR and inflammation as the main common conditions of obesity and AD.

## Figures and Tables

**Table 1:** Metabolic parameters in the APP/PS1 mice and their age-matched controls on STD or HFD at 3, 6, or 10 months of age.

Parameter		Glucose [mmol/L]	Insulin [ng/mL]	Leptin [ng/mL]	Cholesterol [mmol/L]	Triacylglycerol [mmol/L]	FGF21 [pg/mL]	HOMA-IR Index
3 months	WT HFD	7.68 ± 1.95	0.38 ± 0.20	0.49 ± 0.33	5.26 ± 0.43	0.86 ± 0.09	537.53 ± 305.57	23.61 ± 17.02
	APP/PS1 HFD	7.28 ± 1.71	0.38 ± 0.24	0.98 ± 1.10	5.12 ± 1.40	0.85 ± 0.42	900.48 ± 451.98	22.98 ± 16.22
6 months	WT STD	4.96 ± 1.03 **	0.08 ± 0.06 ***	1.18 ± 1.03 ***	3.40 ± 0.32 **	0.55 ± 0.14 **	541.55 ± 338.84	3.24 ± 2.34 *
	WT HFD	6.96 ± 0.97 **	1.36 ± 0.45 ***	55.09 ± 15.00 ***	6.25 ± 1.52 **	0.97 ± 0.27 **	804.94 ± 288.09	73.14 ± 30.44 *
	APP/PS1 STD	4.88 ± 0.59 ****	0.13 ± 0.03 ***	1.72 ± 1.41 ****	3.32 ± 0.79 ****	0.57 ± 0.10 ***	891.75 ± 648.42	4.50 ± 1.06 ***
	APP/PS1 HFD	7.78 ± 0.59 ****	1.95 ± 0.91 ***	67.39 ± 17.41 ****	7.85 ± 1.50 ****	1.23 ± 0.23 ***	1279.26 ± 691.75	117.24 ± 59.40 ***
10 months	WT STD	6.16 ± 0.72	0.27 ± 0.31	1.04 ± 0.79 ****	2.54 ± 1.10 *	0.90 ± 0.35	615.40 ± 253.30	12.35 ± 14.10 **
	WT HFD	7.85 ± 1.40	1.51 ± 1.08 **** ***	67.92 ± 20.86 ****	4.18 ± 1.38 *	1.51 ± 0.50	1013.95 ± 578.83 *	93.48 ± 72.92 ** ***
	APP/PS1 STD	6.32 ± 1.93	0.12 ± 0.08 **** ***	0.78 ± 0.32 ****	2.47 ± 0.49 ***	1.06 ± 0.39 *	824.23 ± 464.01 * *	6.83 ± 6.90 **** ***
	APP/PS1 HFD	6.95 ± 1.24	4.64 ± 1.32 **** ***	86.12 ± 26.20 ****	5.32 ± 1.11 ***	1.65 ± 0.32 *	1999.93 ± 1103.56 * *	254.85 ± 100.81 **** ***

Parameters measured from blood plasma collected from overnight-fasted mice. The data are presented as the means ± SD. Statistical analysis was made between groups fed with STD and HFD as shown in the Table via one-way ANOVA with Bonferroni post hoc test, *: *p* < 0.05, **: *p* < 0.01, ***: *p* < 0.001, ****: *p* < 0.0001 (*n* = 5–12 mice per group). *: Statistical analysis between APP/PS1 and WT mice on HFD HFD *: *p* < 0.05 and ***: *p* < 0.001. HFD: high-fat diet, STD: standard diet, FGF21: fibroblast growth factor 21, HOMA-IR: homeostatic model assessment for insulin resistance.

**Table 2:** Summary of peripheral parameters affected by age, HFD and APP/PS1 genotype.

	Plasma/Tissue	3 Months	6 Months					10 Months				
		APP/PS1 vs. WT on HFD	HFD vs. STD		APP/PS1 vs. WT on HFD	Age 6 M vs. 3 M on HFD		HFD vs. STD		APP/PS1 vs. WT on HFD	Age 10 M vs. 6 M on HFD	
Body Weight		-	↑ ****	↑ ****	-	↑ ^xxxx^	↑ ^xxxx^	↑ ****	↑ ****	↑ ^##^	-	↑ ^xx^
eWAT		-	↑ ****	↑ ****	-	↑ ^xxx^	↑ ^xx^	↑ ***	↑ ****	-	-	↑ ^xx^
Leptin	plasma	-	↑ ***	↑ ****	-	↑ ^xxxx^	↑ ^xxxx^	↑ ****	↑ ****	-	-	-
Glucose	plasma	-	↑ **	↑ ****	-	-	↑ ^xx^	-	-	-	-	-
OGTT AUC	plasma	-	↑ ***	↑ ***	↑ ^###^	-	-	-	-		-	-
Insulin	plasma	-	↑ ***	↑ ***	-	-	↑ ^x^	↑ ***	↑ ***	↑ ^###^	-	↑ ^xxxx^
FGF21	plasma	-	-	-	-	-	-	-	↑ *	↑ ^#^	-	-
HOMA-IR index		-	↑ *	↑ ***	-	-	-	-	-	↑ ^###^	-	↑ ^xxx^
Cholesterol	plasma	-	↑ **	↑ ****	-	-	↑ ^xx^	↑ *	↑ ***	-	-	↓ ^xx^
Triacylglycerol	plasma	-	↑ **	↑ ***	-	-	-	-	↑ *	-	↑ ^x^	↑ ^xx^
CRP	plasma	-	-	↑ *	-	-	-	-	↑ **	-	-	-
LIVER WEIGHT		-	-	↑ **	-	↑ ^x^	↑ ^x^	-	↑ ****	↑ ^###^	-	↑ ^xx^
Steatosis	liver	-	-	↑ **	↑ ^##^	-	-	-	↑ **	-	-	-
Fibrosis	liver	-	-	-	-	-	-	↑ **	↑ ***	-	-	-
IRbeta	liver	-	-	↓ *	-	-	-	-	↓ ***	-	-	-
PI3K	liver	-	↑ **	-	-	-	-	-	-	-	↓ ^xxxx^	↓^xx^
p-Akt (Ser473)	liver	-	-	-	-	-	-	-	-	-	-	-
IRbeta	eWAT	-	↓ ***	↓ ***	-	-	↓ ^xxxx^	↓ *	↓ ***	-	-	-
PI3K	eWAT	-	-	-	-	-	↓ ^xxx^	-	-	-	-	-
p-Akt (Ser473)	eWAT	-	-	-	-	-	-	↓ *	-	-	-	-
GLUT4	eWAT	-	↓ *	-	-	-	-	-	-	-	-	-
IRbeta	muscle	-	-	↓ **	↓ ^##^	-	-	-	↓ *	-	-	-
PI3K	muscle	-	-	↓ **	-	-	-	↓ *	-	-	-	-
p-Akt (Ser473)	muscle	-	↑ **	-	-	-	-	-	-	-	-	-
GLUT4	muscle	-	-	-	-	-	-	↓ *	↓ *	-	-	-

Statistical analysis was made via one-way ANOVA with Bonferroni post hoc test, (*n* = 5–12 mice per group). The significance of changes induced by diet: *: *p* < 0.05, **: *p* < 0.01, ***: *p* < 0.001, ****: *p* < 0.0001, by age: ^x^: *p* < 0.05, ^xx^: *p* < 0.01, ^xxx^: *p* < 0.001, ^xxxx^: *p* < 0.0001, by genotype: #: *p* < 0.05, ##: *p* < 0.01, ###: *p* < 0.001. Difference between the WT mice on HFD and STD signified by blue color, difference between the APP/PS1 mice on HFD and STD signified by red color. ↑: increase; ↓: decrease; HFD: high-fat diet; STD: standard diet; eWAT: epididymal white adipose tissue; OGTT: oral glucose tolerance test; FGF21: fibroblast growth factor 21; HOMA-IR: homeostatic model assessment for insulin resistance; CRP: c-reactive protein.

**Table 3:** Summary of AD-like pathology parameters affected by age, HFD and APP/PS1 genotype.

	Brain Region	3 Months	6 Months					10 Months				
		APP/PS1 vs. WT on HFD	HFD vs. STD		APP/PS1 vs. WT on HFD	Age 6 M vs. 3 M on HFD		HFD vs. STD		APP/PS1 vs. WT on HFD	Age 10 M vs. 6 M HFD	
Aβ plaques	Hippocampus	↑ ^#^	-	-	↑ ^###^	-	-	-	-	↑ ^###^	-	↑ ^xxx^
	Cortex	↑ ^##^	-	-	↑ ^##^	-	↑ ^xxx^	-	↑ *	↑ ^###^	-	↑ ^xxx^
Microgliosis (Iba1)	Hippocampus	-	-	-	↑ ^##^	-	-	-	↑ *	↑ ^###^	-	-
	Cortex	-	-	-	↑ ^##^	-	-	-	↑ *	↑ ^###^	-	-
Astrocytosis (GFAP)	Hippocampus	-	-	-	↑ ^####^	-	↑ ^xx^	-	-	↑ ^##^	-	↑ ^x^
	Cortex	-	-	-	↑ ^##^	-	↑ ^xxxx^	-	-	↑ ^##^	-	↑ ^xx^
Total Tau (9H12)	Hippocampus (CA1)	-	-	-	-	-	-	-	-	-	-	-
p-Tau (Ser202, Thr205) (AT8)	Hippocampus	-	-	↑ *	↑ ^####^	-	-	-	-	↑ ^###^	-	-
	Cortex	-	-	↑ **	↑ ^####^	-	-	-	-	↑ ^###^	-	-
Insulin receptor β	Hippocampus	-	-	-	-	↑ ^xx^	-	-	↓ ****	↓ ^##^	-	↓ ^xxx^
PI3K	Hippocampus	↑ ^##^	-	-	-	-	-	-	↓ *	-	-	-
p-Akt (Ser473)	Hippocampus	-	-	-	-	-	-	-	-	-	-	↓ ^xx^
Neuronal density (NeuN)	Hippocampus	-	-	-	↑ ^##^	↓ ^x^	↓ ^x^	-	-	-	-	-
	Cortex	-	-	-	-	-	↓ ^xx^	-	-	-	-	-
Neurogenesis (DCX)	Hippocampus (DG)	-	-	-	-	↓ ^xxx^	↓ ^xxxx^	-	-	-	-	-
Neurogenesis (Tau 3R)	Hippocampus (DG)	-	-	-	-	↓ ^xxxx^	↓ ^xxxx^	-	-	-	-	-
Synaptophysin	Hippocampus	-	-	-	-	-	-	-	-	↓ ^#^	-	-
Spinophilin	Hippocampus	-	-	-	-	-	-	-	-	-	-	-

Statistical analysis was made via one-way ANOVA with Bonferroni test, (*n* = 5–8 mice per group). The significance of changes induced by diet: *: *p* < 0.05, **: *p* < 0.01, ****: *p* < 0.0001, by age: ^x^: *p* < 0.05, ^xx^: *p* < 0.01, ^xxx^: *p* < 0.001, ^xxxx^: *p* < 0.0001, by genotype: #: *p* < 0.05, ##: *p* < 0.01, ###: *p* < 0.001, ####: *p* < 0.0001. Difference between the WT mice on HFD and STD signified by blue color, difference between the APP/PS1 mice on HFD and STD signified by red color. ↑: increase; ↓: decrease; HFD: high-fat diet; STD: standard diet; CA1: cornu ammonis; DG: dentate gyrus.

## Data Availability

The presented data are available on request from the corresponding authors.

## Supplementary Materials

The following supporting information can be downloaded at: https://www.mdpi.com/article/10.3390/nu15173690/s1, Figure S1: HFD reduced the PI3K/AKT signaling pathway in liver, eWAT and muscle. Figure S2: Decreased neuronal density with age of mice. Figure S3: Decreased neurogenesis in mice with age. Figure S4: HFD decreased synaptogenesis in the hippocampi. Figure S5: PCA score plot based on frontal cortex lipid profiling of WT and APP/PS1 mice on HFD or STD at the age of 10 months. Figure S6: HFD upregulated TGs in the frontal cortex of APP/PS1 mice. Figure S7: MALDI MSI analysis of APP/PS1 mice on HFD and STD at the age of 10 months; Table S1: List of antibodies and their appropriate dilutions used for Western blotting. Table S2: List of antibodies and their appropriate dilutions used for immunohistochemistry. Table S3: HFD-induced significant metabolic changes in urine of APP/PS1 and WT mice at the age of 6 and 10 months. Table S4: Strain-induced significant metabolic changes in urine of APP/PS1 and WT mice at the ages of 6 and 10 months. Table S5: HFD-induced significant metabolic changes in plasma of APP/PS1 and WT mice at the ages of 6 and 10 months. Table S6: Strain-induced significant metabolic changes in plasma of APP/PS1 and WT mice at the ages of 6 and 10 months. Table S7: HFD-induced significant metabolic changes in polar liver extracts of APP/PS1 and WT mice at the age of 10 months. Table S8: Strain-induced significant metabolic changes in polar liver extracts of APP/PS1 and WT mice at the age of 10 months. Table S9: Upregulated liver lipids in the APP/PS1 mice on HFD at the ages of 6 months identified via volcano plot.
