# Supplementary material for: Feeding High-Fat Diet Accelerates Development of Peripheral and Central Insulin Resistance and Inflammation and Worsens AD-like Pathology in APP/PS1 Mice
Source: Nutrients. 2023 Aug 23;15(17):3690. doi: 10.3390/nu15173690 (PMC10490051; doi:10.3390/nu15173690)
Supplement: Supplementary file 1 [file nutrients-15-03690-s001.zip › nutrients-2530557-supplementary.pdf]

Supplementary data

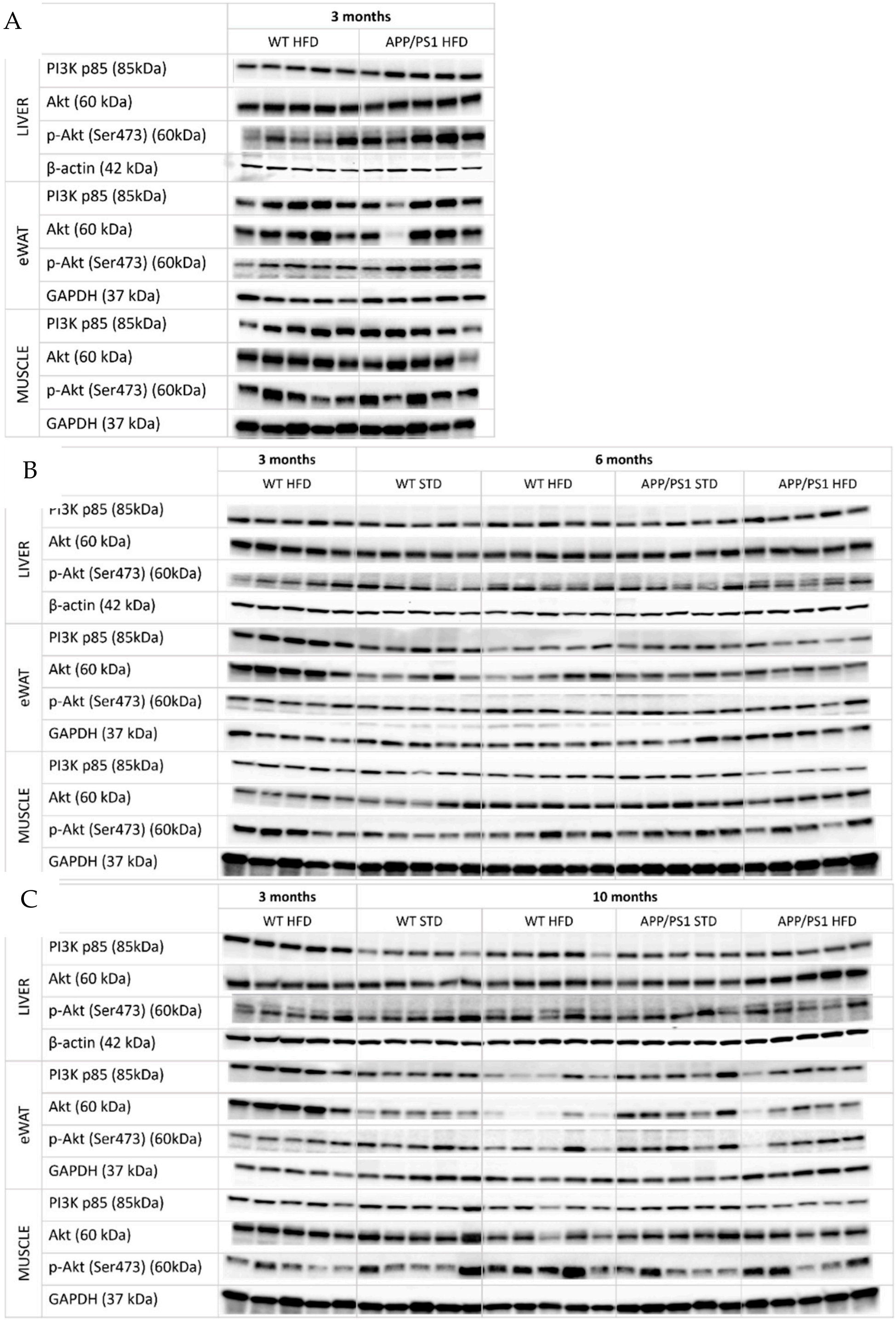

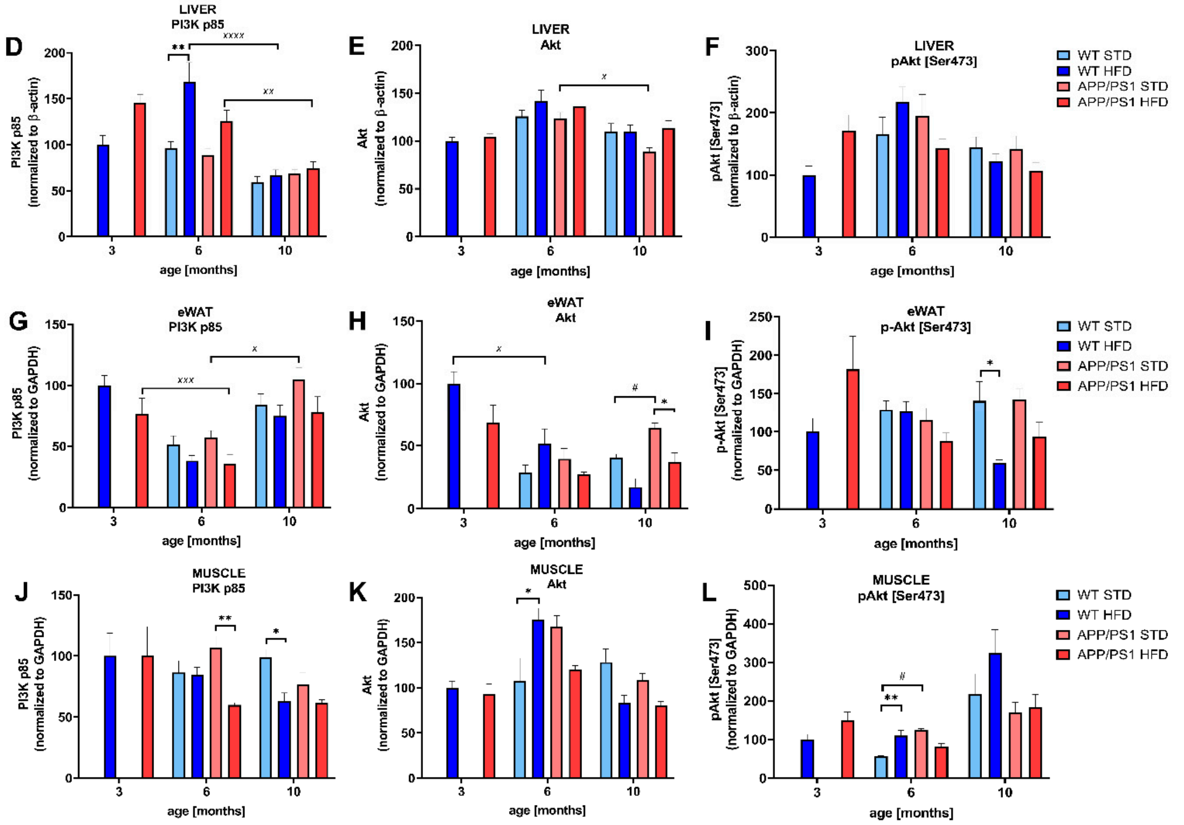

Figure S1: HFD reduced the PI3K/AKT signaling pathway in liver, eWAT and muscle. Proteins were determined by western blotting. Immunoblots in (A) 3 months, (B) 6 months and (C) 10 months of age, (D-L) quantification of protein levels: (D) PI3K p85 (E) Akt and (F) p-Akt (Ser473) in the liver, (G) PI3K p85 (H) Akt and (I) p-Akt (Ser473) in eWAT, (J) PI3K p85 (K) Akt and (L) p-Akt (Ser473) in the skeletal muscle. Percentage of the stained area is expressed as a % of 3-month-old WT on HFD to enable the comparison of multiple staining series. The data are presented as the means ± SEM. Statistical analysis is made by one-way ANOVA with Bonferroni post-hoc test (n = 5-8 mice per group). The significance of changes induced by diet $^{*}p<0.05$, $^{**}p<0.01$, by age $^{x}p<0.05$, $^{xx}p<0.01$, $^{xxx}p<0.001$, $^{xxxx}p<0.0001$, by genotype $^{\#}p<0.05$.

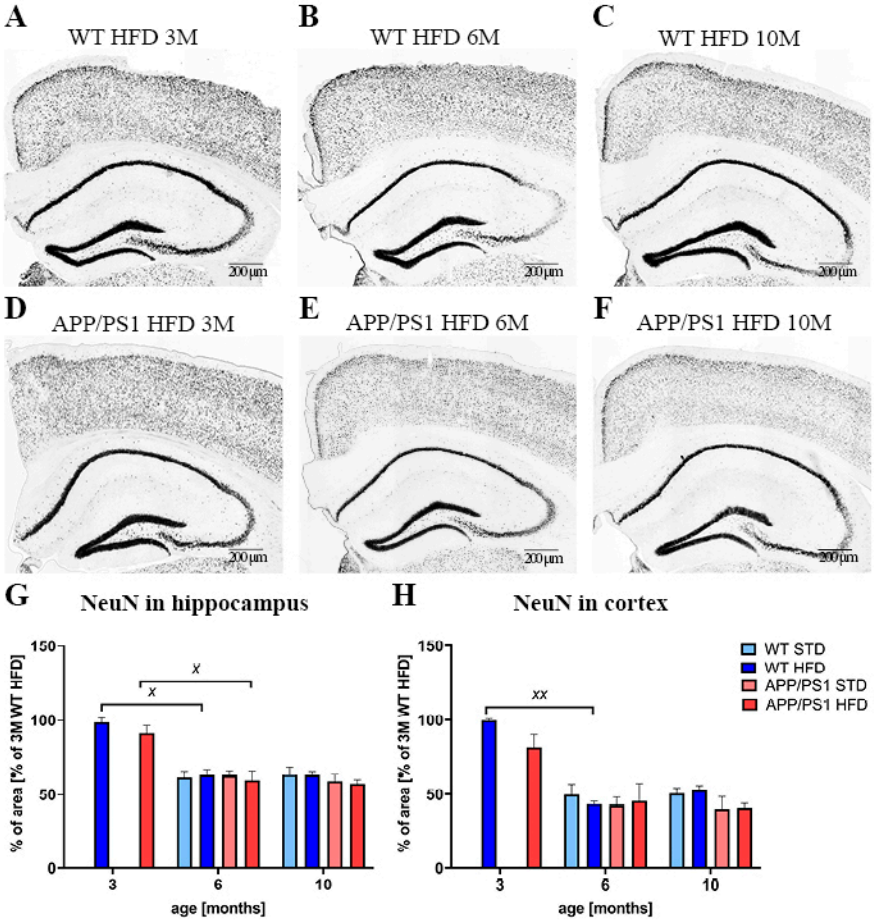

Figure S2: Decreased neuronal density with age of mice. Representative photomicrographs of the WT mice fed with HFD in (A) 3 months, (B) 6 months and (C) 10 months of age and APP/PS1 mice fed with HFD in (D) 3 months, (E) 6 months and (F) 10 months immunohistochemically stained with neuronal marker NeuN, and their quantification (G,H). Percentage of the stained area is expressed as a % of the 3-month-old WT mice on HFD to enable the comparison of multiple staining series. The data are presented as the means ± SEM. Statistical analysis is made by one-way ANOVA with Bonferroni post-hoc test (n = 5-8 mice per group). The significance of changes induced by age $^{x}p<0.05$, $^{xx}p<0.01$.

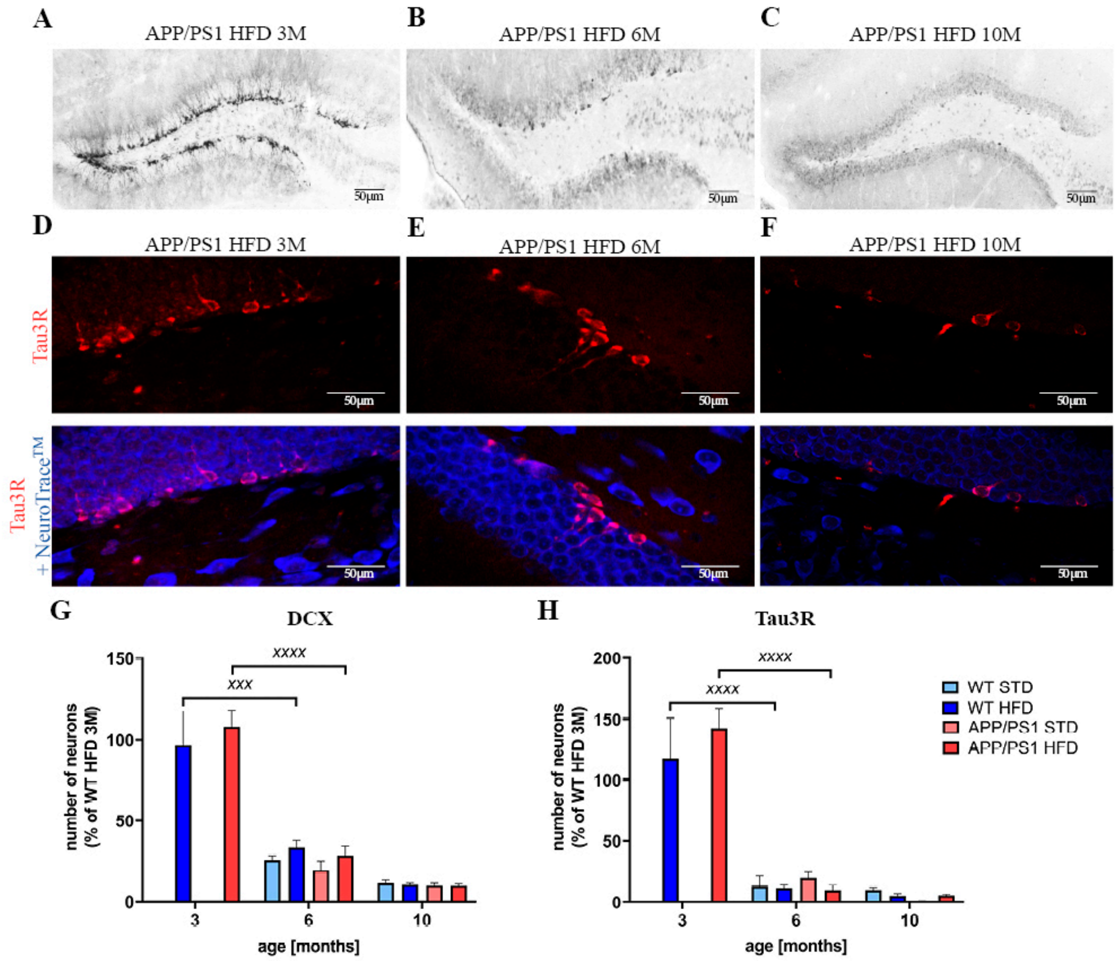

Figure S3: Decreased neurogenesis in mice with age. Representative photomicrographs of the APP/PS1 mice fed with HFD in (A,D) 3 months (B,E) 6 months and (C,F) 10 months of age immunohistochemically stained either for (A-C) doublecortin (DCX), or for (D-F) double staining Tau3R and NeuroTrace™. Percentage of the stained area is expressed as a % of the 3-month-old WT mice on HFD to enable the comparison of multiple staining series. The data are presented as the means ± SEM. Statistical analysis is made by one-way ANOVA with Bonferroni post-hoc test (n = 5-8 mice per group). The significance of changes induced by age $^{xxx}p<0.001$, $^{xxxx}p<0.0001$.

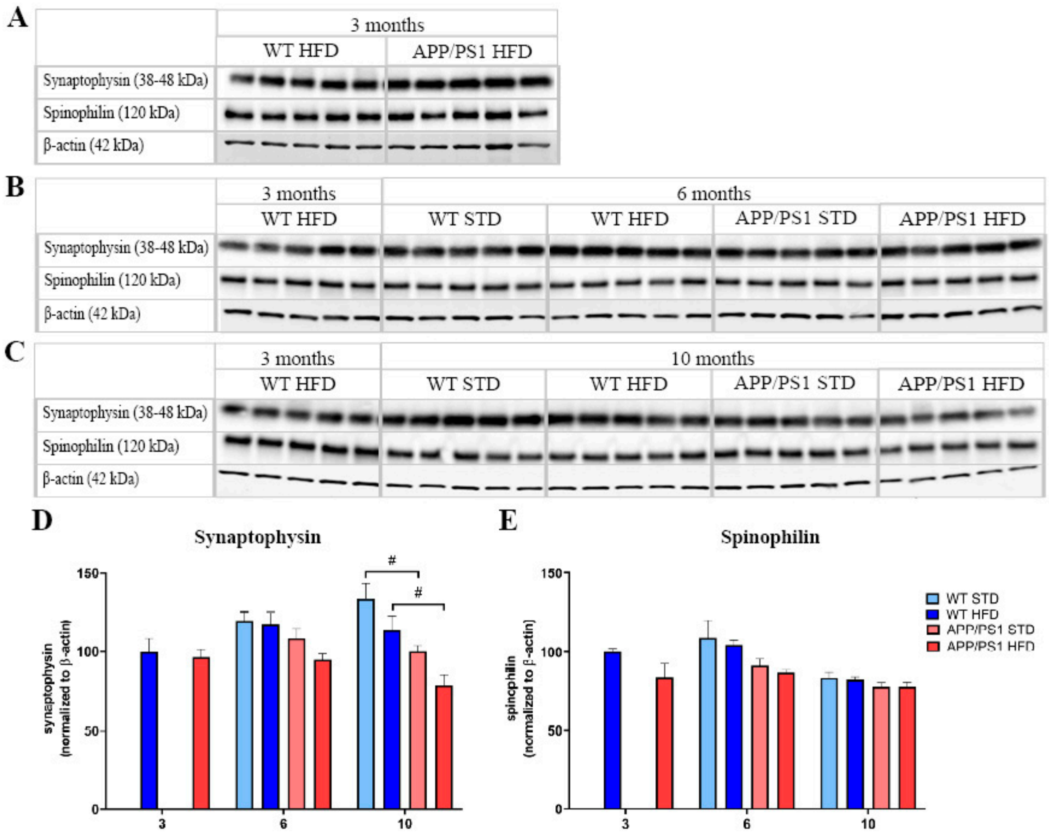

Figure S4: HFD decreased synaptogenesis in the hippocampi. Markers were determined by western blotting. Immunoblots in (A) 3 months, (B) 6 months and (C) 10 months of age, (D-E) quantification of protein levels: (D) synaptophysin (E) spinophilin. The data are presented as the means ± SEM. Statistical analysis is made by one-way ANOVA with Bonferroni post-hoc test (n = 5-8 mice per group). The significance of changes induced by genotype $^{\#}p<0.05$.

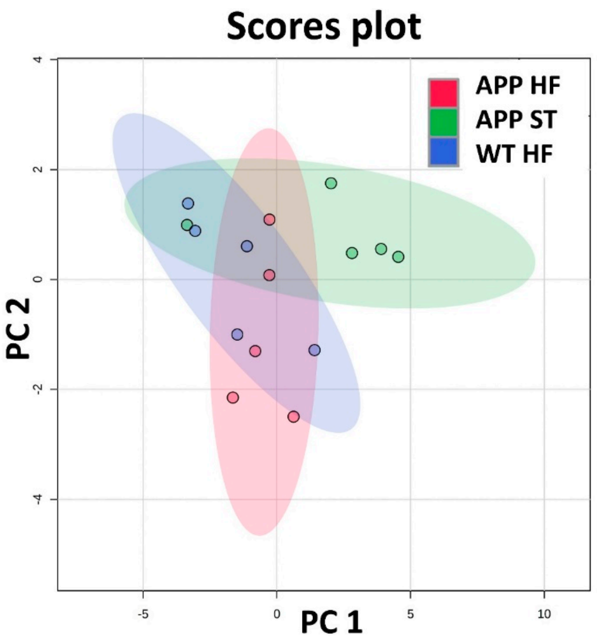

Figure S5: PCA score plot based on frontal cortex lipid profiling of WT and APP/PS1 mice on HFD or STD at the age of 10 months.

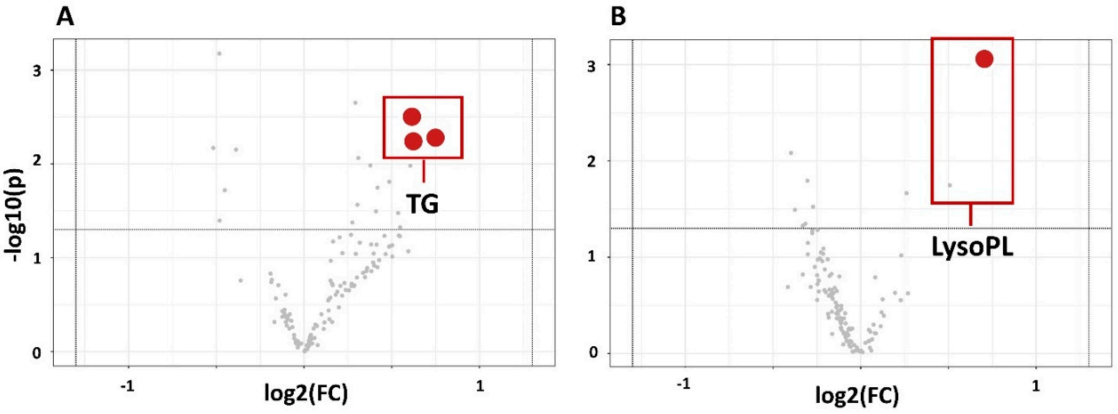

Figure S6: HFD upregulated TGs in the frontal cortex of APP/PS1 mice. Volcano plot of lipids from frontal cortex of APP/PS1 mice on HFD in comparison to STD (A), APP mice on HFD in comparison to WT mice on HFD (B). Statistical analysis is made between groups by Student´s t-test, *p<0.05 (n = 5 mice per group). **TG – different species of triacylglycerols, LysoPL –** lysophosphatidylethanolamine (18:0)**.**

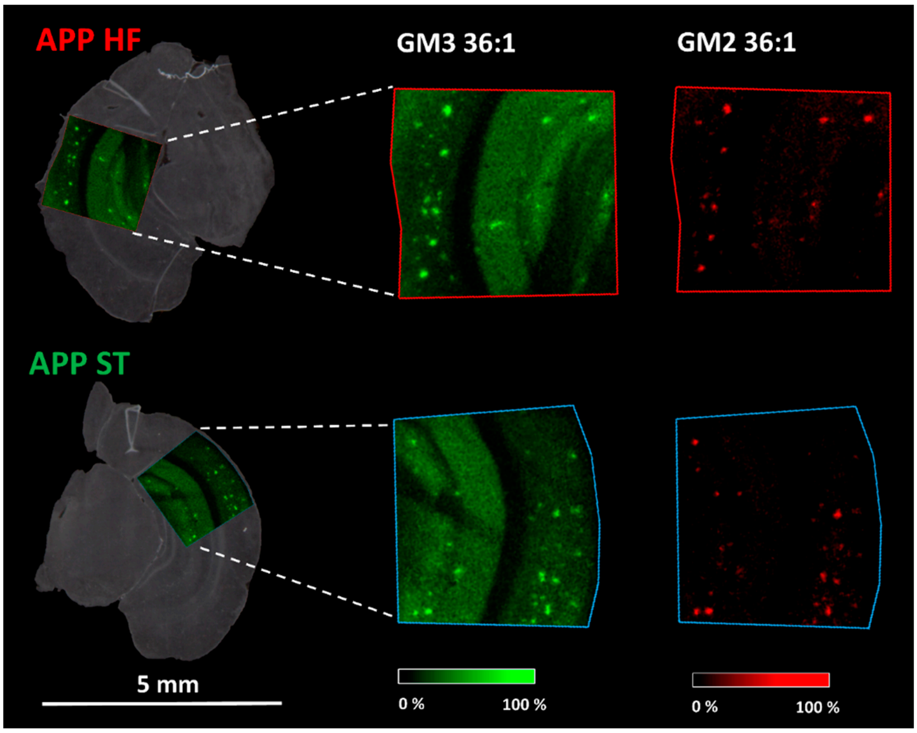

Figure S7: MALDI MSI analysis of APP/PS1 mice on HFD and STD at the age of 10 months. Optical image of coronal brain tissue section with measurement region. Ion images of gangliosides (GM3 36:1, m/z 1179.7 and GM2 36:1, m/z 1382.7) were obtained in negative ion mode ([M - H]-) at spatial resolution 15 µm.

Table S1: List of antibodies and their appropriate dilution used for western blotting.

Antibody	Manufacturer and cat. no.	Blocking	Dilution
Akt, rabbit Ab	Cell Signaling Technology, Beverly, MA, USA, cat. no. 4691	5% milk	1:1 000, 5% BSA
p-Akt (Ser473), rabbit Ab	Cell Signaling Technology, Beverly, MA, USA, cat. no. 4060	5% milk	1:1 000, 5% BSA
GAPDH, mouse Ab	Cell Signaling Technology, Beverly, MA, USA, cat. no. 97166	5% milk	1:1 000, 5% milk
GLUT4, mouse Ab	Cell Signaling Technology, Beverly, MA, USA, cat. no. 2213	5% milk	1:1 000, 5% milk
Insulin receptor β, rabbit Ab	Cell Signaling Technology, Beverly, MA, USA, cat. no. 3025	5% milk	1:1 000, 5% BSA
PI3K p85, rabbit Ab	Cell Signaling Technology, Beverly, MA, USA, cat. no. 4257	5% milk	1:1 000, 5% BSA
PSD95, mouse Ab	Cell Signaling Technology, Beverly, MA, USA, cat. no. 2507	5% BSA	1:1 000, 5% BSA
Synaptophysin, rabbit pAb	Santa Cruz Biotechnology, Dallas, TX, USA, cat. no. sc-9116	5% milk	1:1 000, 5% milk
Spinophilin, rabbit Ab	Cell Signaling Technology, Beverly, MA, USA, cat. no. 14136	5% milk	1:1 000, 5% BSA
β-actin, mouse Ab	Sigma, St. Louis, MO, USA, cat. no. A5441	5% milk	1:10 000, 5% milk

Table S2: List of antibodies and their appropriate dilution used for immunohistochemistry**.**

Antibody	Manufacturer and cat. no.	Dilution
AT8 (phospho-Tau pSer202 + pThr205), mouse mAb	Invitrogen/Thermo Fisher Scientific, Waltham, MA, USA, cat.no. MN1020	1 : 400
β-amyloid, rabbit pAb	Invitrogen/Thermo Fisher Scientific, Waltham, MA, USA, cat. no. 715800	1 : 500
Doublecortin, rabbit pAb	Cell Signaling Technology, Beverly, MA, USA, cat. no. 4604	1 : 600
GFAP, rabbit pAb	Invitrogen/Thermo Fisher Scientific, Waltham, MA, USA, cat. no. PA5-16291	1 : 500
Iba1, rabbit mAb	Fujifilm Wako Pure Chemical Corporation, Osaka, Japan, cat. no. 01919741	1 : 2 000
NeuN, rabbit pAb	Invitrogen/Thermo Fisher Scientific, Waltham, MA, USA, cat.no. 711054	1 : 250
Tau-3R, mouse mAb	Merck Millipore, Burlington, MA, USA, cat.no. 05803	1 : 200
Tau 9H12 (total Tau), mouse mAb	Generous gift from Dr. L. Buée, INSERM, Lille, France	1 : 100

Table S3: HFD-induced significant metabolic changes in urine of APP/PS1 and WT mice at the age of 6 and 10 months.

**effect of diet**	**6 months**				**10 months**			
	**WT (HFD vs STD)**		**APP/PS1 (HFD vs STD)**		**WT (HFD vs STD)**		**APP/PS1 (HFD vs STD)**	
	**Δ [%]**	**p-value**	**Δ [%]**	**p-value**	**Δ [%]**	**p-value**	**Δ [%]**	**p-value**
1-Methylnicotinamide	**103.4**	0.000	**159.7**	0.000	**137.2**	0.000	**172.9**	0.000
2-PY	**104.2**	0.000	**60.7**	0.000	**39.7**	0.004	**67.0**	0.000
Nicotinamide N-oxide	61.6	0.332	125.8	0.065	64.5	0.432	**153.3**	0.024

Trigonelline	-7.7	0.927	-10.1	0.873	**-25.7**	0.013	**-21.7**	0.028
Vinylacetylglycine	**-47.8**	0.000	**-37.2**	0.003	**-40.0**	0.000	**-41.2**	0.000
N-butyrylglycine	**-46.7**	0.000	**-39.8**	0.000	**-30.2**	0.000	**-34.1**	0.000
N-isovalerylglycine	**-33.1**	0.000	**-20.3**	0.000	**-23.0**	0.000	**-28.1**	0.000
Hexanoylglycine	**-44.5**	0.000	**-42.2**	0.000	**-44.7**	0.000	**-44.3**	0.000
Citrate	**-26.9**	0.040	4.2	0.981	**-46.1**	0.014	**-48.8**	0.001
2-Oxoglutarate	**-74.7**	0.000	**-57.7**	0.005	**-77.1**	0.000	**-75.1**	0.000
Fumarate	**-66.5**	0.041	**-66.3**	0.005	**-91.3**	0.000	**-83.7**	0.000
Succinate	-7.7	0.808	**64.8**	0.001	15.9	0.810	6.6	0.981
Glucose + derivatives	**115.7**	0.000	**130.0**	0.000	44.3	0.114	**80.7**	0.001
Unidentified saccharide 1	**145.4**	0.017	**204.5**	0.008	28.7	0.992	192.7	0.109
Unidentified saccharide 2	**55.2**	0.000	**54.3**	0.000	**66.1**	0.003	**54.1**	0.034
Carnitine	**64.9**	0.012	**160.4**	0.000	**126.7**	0.000	**149.5**	0.000
Choline derivative	45.8	0.159	**101.5**	0.003	**253.1**	0.000	**139.6**	0.003
Methylamine	**-47.0**	0.000	**-42.2**	0.000	**-44.6**	0.000	**-39.1**	0.000
Trimethylamine	-17.5	0.622	**-65.6**	0.000	**-47.7**	0.001	**-57.6**	0.000
N-carbamoyl β-alanine	**117.7**	0.000	**65.3**	0.001	**106.0**	0.000	**148.3**	0.000
Creatinine	**24.4**	0.003	**51.5**	0.000	**38.4**	0.000	**52.7**	0.000
Glycine	**34.7**	0.031	**69.9**	0.000	34.9	0.097	**40.1**	0.048
Taurine	**-69.7**	0.000	**-71.9**	0.000	**-48.3**	0.023	**-46.0**	0.003
Hippurate	-13.6	0.730	0.7	1.000	**-43.0**	0.009	**-37.5**	0.009
Putrescine	-15.8	0.172	-5.1	0.911	**-32.7**	0.004	-19.7	0.190
Orotate	**54.1**	0.004	28.3	0.161	0.4	1.000	2.5	0.997
2-Oxoisocaproate	**-38.6**	0.000	**-24.6**	0.007	**-25.7**	0.001	**-24.8**	0.006
3-Methyl-2-oxovalerate	**-37.4**	0.000	**-27.1**	0.001	**-43.7**	0.013	**-31.0**	0.041
Pseudouridine	-0.4	1.000	**17.7**	0.050	**24.2**	0.001	**22.2**	0.007
Methanol	13.0	0.882	9.2	0.953	**65.7**	0.009	17.6	0.780

The results are expressed as the percentage change of concentration in HFD vs. STD mice of both strains. The statistical significance was analysed by parametric ANOVA. Significant changes with p<0.05 are color-coded: significant increase in red, decrease in blue. HFD - high-fat diet, STD – standard diet, 2PY - N-Methyl-2-pyridone-3-carboxamide.

Table S4: Strain-induced significant metabolic changes in urine of APP/PS1 and WT mice at the age of 6 and 10 months.

**effect of strain**	**6 months**				**10 months**			
	**STD** (APP/PS1 vs WT)		**HFD** (APP/PS1 vs WT)		**STD** (APP/PS1 vs WT)		**HFD** (APP/PS1 vs WT)	
	**Δ [%]**	**p-value**	**Δ [%]**	**p-value**	**Δ [%]**	**p-value**	**Δ [%]**	**p-value**
2-PY	**-15.6**	0.006	-5.2	0.303	-4.5	0.948	-0.6	0.999
Unidentified saccharide 1	28.5	0.969	3.6	0.996	-22.3	0.995	**-65.8**	0.018
Glucose + derivatives	19.2	0.887	11.8	0.579	-3.8	0.998	**-23.1**	0.032
Creatinine	6.5	0.802	**-12.6**	0.014	17.1	0.112	6.2	0.202
Carnitine	12.8	0.949	**-28.6**	0.003	-5.9	0.997	-14.5	0.224
Trimethylamine	**-35.0**	0.014	56.0	0.118	-9.2	0.875	12.0	0.915
Succinate	**64.1**	0.003	-8.1	0.705	-6.8	0.987	1.3	1.000

3-Methyl-2-oxovalerate	-2.6	0.982	-16.4	0.163	-21.3	0.359	-35.8	0.025
N-isovalerylglycine	-2.1	0.956	-17.8	0.001	-14.2	0.054	-8.1	0.326

The results are expressed as the percentage change of concentration in APP/PS1 vs. WT mice on both diets. The statistical significance was analysed by parametric ANOVA. Significant changes with $p<0.05$ are color-coded: significant increase in red, decrease in blue. HFD - high-fat diet, STD – standard diet, 2PY - N-Methyl-2-pyridone-3-carboxamide.

Table S5: HFD-induced significant metabolic changes in plasma of APP/PS1 and WT mice at the age of 6 and 10 months.

effect of diet	6 months				10 months			
	WT (HFD vs STD)		APP/PS1 (HFD vs STD)		WT (HFD vs STD)		APP/PS1 (HFD vs STD)	
	Δ [%]	p-value	Δ [%]	p-value	Δ [%]	p-value	Δ [%]	p-value
Glucose	36.0	0.021	37.5	0.004	4.0	0.986	25.4	0.222
Arabinose	5.5	0.952	36.3	0.014	-4.0	0.976	23.4	0.174
Valine	-45.2	0.025	-46.3	0.008	-17.6	0.310	-22.0	0.239
Leucine	-41.7	0.056	-45.5	0.012	-19.0	0.168	-16.1	0.416
Phenylalanine	-26.4	0.040	-24.7	0.046	-9.7	0.492	-10.7	0.420
2-Hydroxyisobutyrate	-46.3	0.002	-17.8	0.414	-40.0	0.923	-14.8	0.986
3-Hydroxyisobutyrate	-58.9	0.001	-61.8	0.000	-41.9	0.094	**-52.1**	0.044
2-Oxoisocaproate	-33.9	0.006	-26.2	0.062	-9.3	0.679	-4.8	0.952
3-Methyl-2-oxovalerate	-39.0	0.010	-21.4	0.527	1.5	1.000	-3.3	0.995
Taurine	-35.2	0.002	-15.4	0.355	-11.8	0.617	-31.4	0.021
Creatine	-32.8	0.010	-6.4	0.919	-11.4	0.827	-31.6	0.032
Dimethylamine	-35.2	0.001	-21.8	0.060	-12.4	0.717	-4.2	0.986
Glycine	-4.5	0.965	-10.2	0.610	-6.7	0.837	-23.2	0.044
Dimethylglycine	50.4	0.007	62.9	0.007	-4.0	0.992	14.9	0.923
Glycerol	12.3	0.795	59.4	0.008	25.9	0.343	17.0	0.655

The results are expressed as the percentage change of concentration in HFD vs. STD mice of both strains. The statistical significance was analysed by parametric ANOVA. Significant changes with $p<0.05$ are color-coded: significant increase in red, decrease in blue. HFD - high-fat diet, STD – standard diet.

Table S6: Strain-induced significant metabolic changes in plasma of APP/PS1 and WT mice at the age of 6 and 10 months.

effect of strain	6 months				10 months			
	STD (APP/PS1 vs WT)		HFD (APP/PS1 vs WT)		STD (APP/PS1 vs WT)		HFD (APP/PS1 vs WT)	
	Δ [%]	p-value	Δ [%]	p-value	Δ [%]	p-value	Δ [%]	p-value
Arabinose	-3.6	0.989	24.6	0.044	-8.7	0.872	17.4	0.194
Lactate	40.9	0.305	21.2	0.295	-3.3	1.000	67.1	0.023
3-Hydroxybutyrate	-23.1	0.848	-23.3	0.520	-18.3	0.843	-46.1	0.027
Alanine	18.8	0.708	16.9	0.715	-5.9	0.990	48.7	0.014

The results are expressed as the percentage change of concentration in APP/PS1 vs. WT mice on both diets. The statistical significance was analysed by parametric ANOVA. Significant changes with $p<0.05$ are color-coded: significant increase in red, decrease in blue. HFD - high-fat diet, STD – standard diet.

Table S7: HFD-induced significant metabolic changes in polar liver extracts of APP/PS1 and WT mice at the age of 10 months.

effect of diet	APP/PS1 (HFD vs STD)		WT (HFD vs STD)	
	Δ [%]	p-value	Δ [%]	p-value
Aspartate	**-45.3**	0.040	-14.5	0.830
Glycine	**-29.5**	0.004	-11.5	0.553
Valine	**-23.4**	0.037	-1.1	0.999
Arabinose	**97.7**	0.013	85.8	0.092
Glucose	**81.4**	0.017	68.5	0.122
Glycogen	**357.1**	0.003	158.2	0.238
Mannose	**61.5**	0.030	65.9	0.056
Hypoxanthine	**-31.4**	0.001	2.1	0.996
Nicotinamide	**-22.7**	0.027	-0.9	1.000
NAD^+	**-34.3**	0.000	-14.9	0.219
Xanthosine	**-28.6**	0.025	**-24.0**	0.047
Uridine	**-28.7**	0.001	-6.6	0.845
Taurine	**-31.6**	0.000	-7.8	0.780
sn-Glycero-3-phosphocholine	**172.0**	0.003	59.1	0.381
Lactate	**75.0**	0.000	37.8	0.114
Glycerol	**-28.1**	0.029	-6.9	0.917

The results are expressed as the percentage change of concentration in HFD vs. STD mice of both strains. The statistical significance was analysed by parametric ANOVA. Significant changes with $p<0.05$ are color-coded: significant increase in red, decrease in blue. HFD - high-fat diet, STD – standard diet, NAD - nicotinamide adenine dinucleotide.

Table S8: Strain-induced significant metabolic changes in polar liver extracts of APP/PS1 and WT mice at the age of 10 months.

effect of strain	STD (APP/PS1 vs WT)		HFD (APP/PS1 vs WT)	
	Δ [%]	p-value	Δ [%]	p-value
Glutamate	**-17.4**	0.024	1.8	0.996
Valine	**-19.0**	0.041	4.6	0.966
Hypoxanthine	-11.9	0.397	**31.2**	0.046
Lactate	**29.0**	0.023	1.6	1.000

The results are expressed as the percentage change of concentration in APP/PS1 vs. WT mice on both diets. The statistical significance was analysed by parametric ANOVA. Significant changes with $p<0.05$ are color-coded: significant increase in red, decrease in blue. HFD - high-fat diet, STD – standard diet.

Table S9: Upregulated liver lipids in the APP/PS1 mice on HFD at the age of 6 months identified by volcano plot.

Lipid	FC	log2(FC)	raw p-value	-log10(p)
DG(16:0_18:1)	2.0581	1.0413	0.001728	2.7625
TG(16:0_16:1_18:1)	2.9308	1.5513	0.0040256	2.3952
TG(16:0_18:1_18:2)	2.0378	1.027	0.0040259	2.3951
TG(20:0_16:0_18:1)	2.639	1.4	0.0047198	2.3261
TG(16:0_18:1_16:0)	2.9313	1.5515	0.0047395	2.3243
TG(18:0_17:0_18:1)	2.3842	1.2535	0.0061069	2.2142
TG(18:1_18:1_16:0)	2.3603	1.239	0.0071435	2.1461
TG(16:0_17:0_18:1)	2.8003	1.4856	0.0071752	2.1442
TG(18:0_18:1_18:1)	2.0426	1.0304	0.0071999	2.1427
TG(16:1_16:1_18:1)	2.3593	1.2384	0.011332	1.9457
TG(20:1_18:1_16:0)	2.7517	1.4603	0.011479	1.9401
TG(18:1_17:1_16:1)	2.0755	1.0535	0.012758	1.8942
TG(18:1_17:1_18:1)	2.1221	1.0855	0.014064	1.8519
TG(18:1_18:1_18:1)	2.0195	1.014	0.015506	1.8095
TG(16:0_18:1_20:4)	2.0438	1.0313	0.015882	1.7991
TG(16:0_16:1_16:1)	2.7149	1.4409	0.015962	1.7969
TG(20:1_18:1_20:1)	2.5052	1.3249	0.01831	1.7373
TG(20:1_18:1_18:1)	2.6005	1.3788	0.01991	1.7009
TG(16:1_16:1_18:2)	2.0024	1.0018	0.022634	1.6452

Statistical analysis is made by Student´s t-test, $p<0.05$. DG – diacylglycerol, TG – triacylglycerol, FC – fold change
